# MnUA–DOX–artesunate hydrogel remodels immunosuppressive tumor microenvironment and prevents postoperative recurrence in triple-negative breast cancer

**DOI:** 10.1186/s12951-026-04381-7

**Published:** 2026-04-18

**Authors:** Keneng Cai, Run Xia, Mengyao Xu, Wanying Chen, Wenli Cai, Xinyu Liu, Tiantian Guo, Weichi Jiang, Chuyi Yu, Jianjia Feng, Chengli Ling, Sheng Zhou, Yinhuan Chen, Changsheng Deng, Qin Xu, Jianming Liang

**Affiliations:** 1 Artemisinin Research Center, Guangzhou University of Chinese Medicine, Guangzhou, 510006 China; 2 The First Afffliated Hospital of Guangzhou University of Chinese Medicine, Guangzhou, 510405 China; 3 Hunan academy of Chinese Medicine, Yuehua road, Yuelu district, Changsha, 410013 China

## Abstract

Triple-negative breast cancer (TNBC) remains a formidable clinical challenge because of its high aggressiveness, metastatic potential, and lack of effective therapeutic options. In this study, we developed an injectable multifunctional hydrogel, MND-ART-GEL, by encapsulating immunostimulatory manganese urate (MnUA), the chemotherapeutic agent doxorubicin (DOX), and an artemisinin derivative, artesunate (ART), within a thermosensitive Pluronic F127 matrix. Uric acid crystals (MSU) and manganese ions (Mn^2+^) were coordinated and assembled into manganese urate (MnUA). The MSU–Mn^2+^ system exhibits a synergistic immunoactivating effect by combining the DAMP-like immune stimulation of MSU with the STING pathway activation ability of Mn^2+^. Owing to its simple chemical composition, low cost, and accessibility, this combination offers a more practical and scalable alternative to conventional synthetic immune agonists. ART synergizes with DOX to exert potent antitumor effects. MND-ART-GEL synergistically elevated intracellular ROS levels, inducing oxidative stress, mitochondrial damage, and subsequent tumor cell apoptosis, immunogenic cell death (ICD), and exposure to immune markers such as MHCI and Fas. Moreover, MND-ART-GEL downregulated STAT3 expression, thereby suppressing tumor cell invasion and migration. In orthotopic and postsurgical TNBC mouse models, MND-ART-GEL significantly inhibited tumor growth and recurrence, remodeled the immunosuppressive tumor microenvironment, enhanced CD8^+^ T-cell infiltration, dendritic cell maturation, and M1 macrophage polarization, reduced PD-1/PD-L1 expression on CD8^+^ T cells, and promoted the secretion of immune-related cytokines. This study presents a localized drug delivery strategy that integrates chemotherapy, immune modulation, and multitarget synergy, offering a promising approach to overcome therapeutic resistance and reduce recurrence in TNBC.

**Graphical Abstract:**

**Figure:**
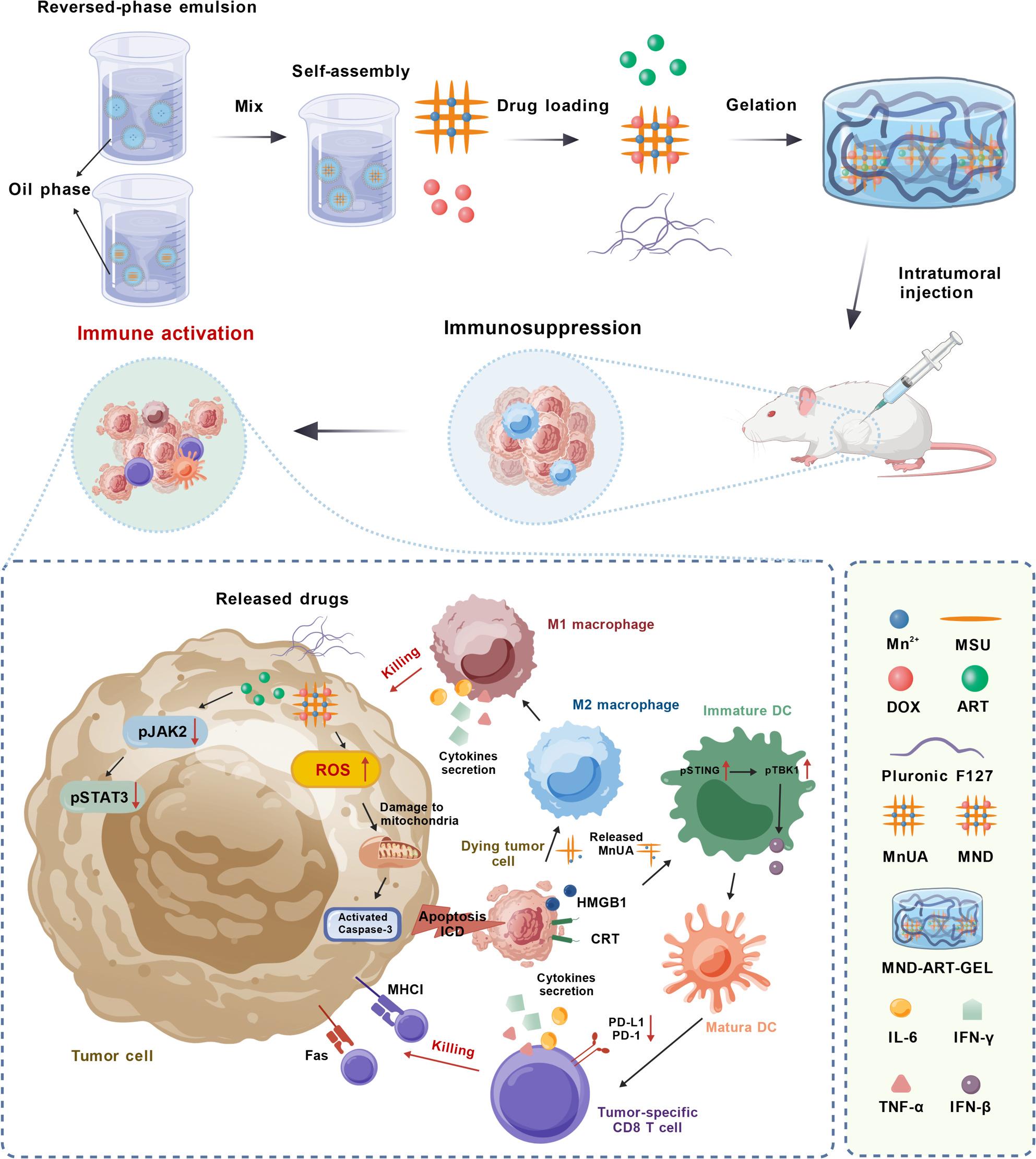
Schematic illustration of the formation of MND-ART-GEL and its intratumoral delivery, highlighting its synergistic chemo-immunotherapeutic mechanism in TNBC through ROS-mediated tumor cell apoptosis, ICD induction, STAT3 inhibition, and remodeling of the tumor immune microenvironment.

**Supplementary Information:**

The online version contains supplementary material available at 10.1186/s12951-026-04381-7.

## Introduction

Breast cancer is one of the most common cancers and the second leading cause of cancer-related deaths among women worldwide [1]. In 2022, approximately 2.3 million new cases of breast cancer were reported, and 670,000 breast cancer-related death occurred globally [2]. Triple-negative breast cancer (TNBC), a breast cancer subtype characterized by the absence of estrogen receptor (ER), progesterone receptor (PR), and human epidermal growth factor receptor 2 (HER2), lacks specific therapeutic targets [3]. Consequently, endocrine therapy and HER2-targeted drugs are ineffective in patients with TNBC. Moreover, TNBC is highly aggressive, prone to metastasis, and has a narrow therapeutic window [4]. Chemotherapy remains the primary treatment for TNBC; however, it often leads to drug resistance and significant adverse effects upon prolonged administration at high doses [5, 6]. Although a subset of patients with TNBC responds to immunotherapy, such as programmed cell death ligand 1/programmed cell death protein 1 (PD-L1/PD-1) blockade, the high heterogeneity of TNBC results in many patients exhibiting immunosuppressive “cold tumor” phenotypes, rendering immunotherapy less effective [7, 8]. Surgical resection is the cornerstone of potential cure for early stage TNBC; however, postoperative recurrence is frequent and poses a serious threat. Clinical data indicate that 30–40% of patients with TNBC relapse within five years of surgery, with poor prognosis and high metastatic potential [9]. Recurrent TNBC tumors are often resistant to chemotherapy, with a predominantly suppressive immune microenvironment that limits the efficacy of immunotherapy [10]. These challenges highlight the inadequacy of monotherapies and underscore the urgent need to develop novel therapeutic strategies. Given the limited efficacy of single-modality treatments, combination therapeutic strategies have emerged as promising approaches for TNBC management [11–13]. In particular, the rational integration of chemotherapy with immune modulation has gained increasing attention for its potential to simultaneously induce tumor cell death and reshape the immunosuppressive tumor microenvironment [14]. Furthermore, considering the high recurrence rate following surgical resection, localized postoperative interventions capable of eliminating residual tumor cells and reprogramming the tumor bed are highly desirable [15, 16]. Injectable thermosensitive hydrogels offer a clinically attractive platform, because they undergo sol–gel transition at physiological temperatures and form an in situ drug depot at the surgical site, enabling sustained local delivery while minimizing systemic exposure [17].

Manganese ions (Mn²^+^), as natural cGAS agonists, induce robust immune responses by activating the cGAS-STING pathway [18]. This is indicative of their potential as immune adjuvants capable of modulating cellular immunity, which can be leveraged to overcome the limitations of traditional aluminum-based adjuvants, which predominantly elicit humoral immunity [19]. Unlike immune checkpoint inhibitors, such as PD-1/PD-L1 blockers, which mainly enhance T cell function, Mn²^+^ can directly reprogram the tumor microenvironment (TME) by promoting the maturation and differentiation of tumor-associated dendritic cells (DCs) and stimulating substantial production of type I interferon, thereby enhancing the infiltration of CD8^+^ T cells into tumors [20–22]. This effectively reverses the immunosuppressive TME and converts poorly immunogenic “cold tumors” into immune-responsive “hot tumors.”

Recent evidence indicates that NF-κB activation prevents the intracellular degradation of activated STING, thereby amplifying STING-associated signaling cascades [23]. As a key regulator of immune homeostasis, NF-κB plays a crucial role in coordinating innate and adaptive immune responses. NF-κB signaling can be triggered by Toll-like receptors (TLRs), whereas monosodium urate (MSU) crystals, which act as a typical damage-associated molecular pattern (DAMP), can activate TLRs/NF-κB [24]. These findings suggest that MSU enhances Mn^2+^-induced immune activation, highlighting its potential as an adjuvant for antitumor immunotherapy. Importantly, both MSU and Mn^2+^ are common endogenous or inorganic components that are widely available, easily prepared, and inexpensive, offering excellent accessibility and scalability. Compared with other expensive or synthetically demanding immune activators, this combination exhibits superior economic and translational advantages, making it highly promising for large-scale preparation and clinical application.

Artesunate (ART), an artemisinin derivative, has been extensively investigated for its antitumor potential in recent years. The STAT3 pathway, which is highly activated in TNBC, plays a pivotal role in promoting tumor proliferation, metastasis, and immune evasion [25, 26]. ART can inhibit the phosphorylation and nuclear translocation of STAT3 [27], thereby disrupting the positive feedback loop between STAT3 and IL-6/JAK2 in various tumor models, including colon and liver cancers [28, 29]. ART suppresses tumor stemness and reverses the immunosuppressive TME. Moreover, it promotes mitochondrial damage via reactive oxygen species (ROS) accumulation [30] and synergizes with doxorubicin (DOX) to exert potent antitumor effects [31]. These properties make ART a promising candidate for overcoming chemoresistance.

Therefore, in this study, we propose a novel manganese urate immunomodulatory material (MnUA) formed through stable coordination-driven self-assembly between manganese ions and urate anions, rather than a simple physical mixture of monosodium urate (MSU) and Mn^2+^. Compared with MSU or Mn^2+^ alone, MnUA structurally integrates the pattern recognition properties of MSU with the innate immune–enhancing capacity of Mn^2+^, thereby endowing the material with a synergistically amplified immunoactivation mode and stronger and more sustained immunomodulatory potential.

**Scheme 1:**
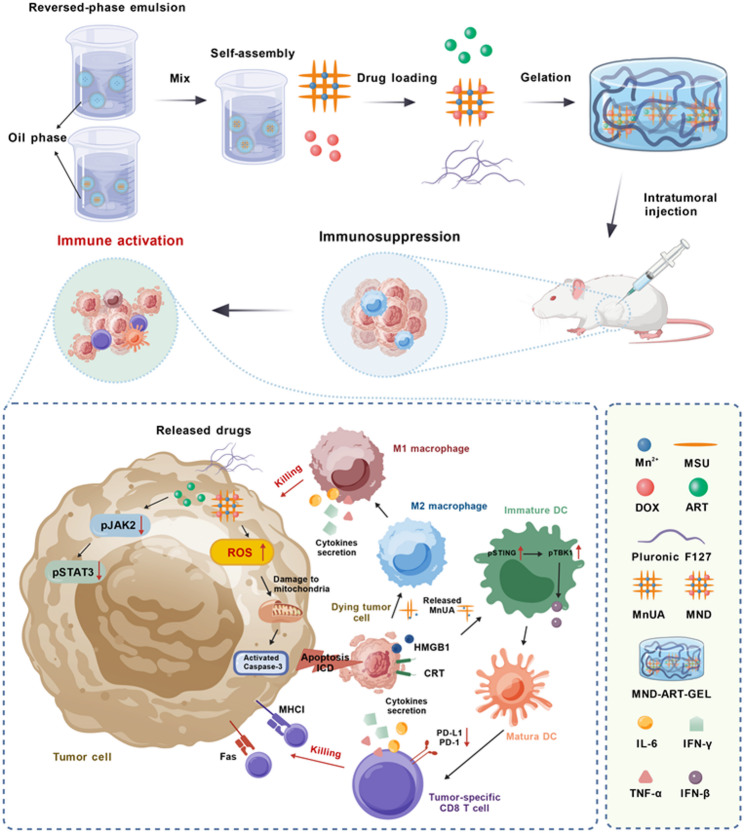
Schematic illustration of the anti-tumor mechanism of MND-ART-GEL. Created with BioGDP.com [51]

Based on this, MnUA was co-loaded with the chemotherapeutic agents doxorubicin hydrochloride and artesunate into a Pluronic F127 thermosensitive hydrogel to construct a localized delivery platform that integrates chemotherapy, immunomodulation, and STAT3 signaling downregulation to improve therapeutic efficacy against triple-negative breast cancer (TNBC) (Scheme 1). This strategy aims to suppress the high invasiveness and metastatic potential of TNBC, reverse the immunosuppressive tumor microenvironment, and enhance therapeutic responses in both primary tumor and postoperative recurrence models.

Notably, both MSU and Mn^2+^ are widely available, involve simple synthetic routes, are inexpensive, and do not rely on complex organic synthesis or biomacromolecular modification, which confers MnUA with clear feasibility and economic advantages for large-scale preparation and practical applications. In the context of current STING-related immunotherapeutic strategies, which largely depend on highly engineered agonists, this study provides a more straightforward and practically viable alternative.

## Methods

### Preparation of MnUA

MnUA particles were prepared using a reverse microemulsion technique. Briefly, 10 mL of cyclohexane, 2 mL of Triton X-100, and 1 mL of *n*-hexanol were mixed in two separate glass vials and stirred vigorously for 10 min. Thereafter, 1 mL of 2 mg/mL MSU suspension in phosphate-buffered saline (PBS) was added to one vial (MSU phase), and 1 mL of 4 mg/mL MnCl_2_ aqueous solution was added to the other vial (Mn phase). The contents of each vial were stirred vigorously for 30 min. The two phases were then combined and stirred vigorously for 1 h to allow the reaction to proceed. The organic solvent was then removed by rotary evaporation. An equal volume of absolute ethanol was added to break the emulsion, followed by centrifugation at 10,000 rpm for 10 min to collect the precipitate. The precipitate was washed once with absolute ethanol and twice with ultrapure water, resuspended in an appropriate amount of ultrapure water, and stored at 4 °C.

### Preparation of MnUA-DOX (MND)

MnUA particles were resuspended in a 2 mg/mL aqueous solution of doxorubicin hydrochloride and incubated at 37 °C for 6–12 h with shaking at 100 rpm. Incubation was terminated when the particles turned dark red, indicating successful drug loading. The resulting MND particles were collected via centrifugation at 10,000 rpm for 10 min. The supernatant was collected to determine the drug loading efficiency.

### Preparation of MND-ART-GEL

ART was weighed at eight times the mass of DOX in MND. ART and Pluronic F127 were dissolved in absolute ethanol at a mass ratio of 1:5. Ethanol was evaporated at 37 °C to form a thin film, which was then hydrated using an appropriate volume of PBS with ultrasonication. After centrifuging 1 mL of the MND suspension at 10,000 rpm to remove the supernatant, 1 mL of the hydrated ART/F127 suspension was added. The mixture was probe-sonicated in an ice bath at 200 W for 5 min to ensure uniform dispersion of ART and MND. The final concentration was adjusted to 22% using Pluronic F127. The mixture was stirred overnight at 4 °C to ensure complete dissolution, yielding the final thermosensitive hydrogel formulation, which was designated “MND-ART-GEL.”

#### Scanning Electron Microscopy (SEM)

Morphology of the hydrogels was observed using a scanning electron microscope (Phenom ProX, Thermo Fisher Scientific, USA). The Samples were directly mounted on the cryostage for imaging.

#### Fourier Transform Infrared Spectroscopy (FTIR)

FTIR spectra were recorded using a Cary 630 FTIR spectrometer (Agilent Technologies, USA) over the range of 4000–400 cm⁻¹ to analyze the functional groups and coordination interactions.

#### X-ray Photoelectron Spectroscopy (XPS)

XPS analysis was performed using a Thermo Scientific K-Alpha spectrometer (Thermo Fisher Scientific, USA) to determine the elemental composition and chemical states.

#### X-ray Diffraction (XRD)

XRD patterns were collected using a Bruker D8 Venture diffractometer (Bruker, Germany) to evaluate the crystalline structure.

#### Drug Release

DOX solution, ART solution, MND, and MND-ART-GEL were prepared separately and adjusted to the same DOX concentration. For each group, 1 mL of the formulation was loaded into a dialysis bag (molecular weight cut-off: 14000 Da) with both ends tightly sealed. The dialysis bags were immersed in 100 mL of PBS and incubated at 37 °C on a shaker at 100 rpm. At predetermined time points (0.5, 1, 2, 3, 4, 5, 6, 8, 10, 12, 24, 48, 72, 96, 120, and 168 h), 1 mL of the release medium was withdrawn and immediately replenished with 1 mL of fresh PBS. The collected samples were filtered through a 0.22 μm membrane filter and analyzed using high-performance liquid chromatography (HPLC). DOX was analyzed using a C18 column (150 × 4.6 mm, 5 μm) with a mobile phase of acetonitrile/0.1% phosphoric acid (30:70, v/v) at a flow rate of 1.0 mL/min, 25 °C, and a detection wavelength of 480 nm (injection volume: 20 µL). ART was analyzed under identical chromatographic conditions, except that the mobile phase ratio was 45:55 (v/v), and detection was performed at 210 nm. The cumulative release percentages of DOX and ART were calculated and plotted as release profiles over time.

#### Cell Culture

4T1 (mouse triple-negative breast cancer cells), RAW264.7 (mouse monocyte-macrophage leukemia cells), and HL-1 (murine cardiomyocytes) were obtained from the Cell Bank of the Chinese Academy of Sciences (Shanghai, China). 4T1 cells were cultured in RPMI-1640 medium supplemented with 10% fetal bovine serum (FBS), 100 U/mL penicillin, and 100 µg/mL streptomycin at 37 °C in a humidified 5% CO_2_ incubator. RAW264.7 cells were cultured in DMEM, and HL-1 cells were cultured in DMEM/F12, both supplemented with 10% FBS, 100 U/mL penicillin, and 100 µg/mL streptomycin, under identical conditions. All experiments were performed using cells within 15 passages.

#### Cytotoxicity Assay

cytotoxicity was assessed using the Cell Counting Kit (CCK)-8 assay. 4T1 cells were seeded in 96-well plates at a density of 5 × 10³ cells per well and incubated overnight. The medium was then replaced with a drug-containing medium at MND: ART mass ratios of 1:2, 1:4, 1:6, 1:8, and 1:10. The MND dosage was calculated based on the DOX concentration, and the final DOX concentrations were set at 2000, 1000, 500, 250, 125, 62.5, 31.25, 15.625, 7.813, and 0 ng/mL. After 48 h of incubation, CCK-8 solution was added to each well and incubated for an additional 1 h. Absorbance at 450 nm was measured using a microplate reader (Varioskan LUX; Thermo Fisher, USA). Half-maximal inhibitory concentration (IC_50_) values were calculated using GraphPad Prism software (version 10). The combination index (CI) at the IC_50_ level was determined using Compusyn software based on the Chou–Talalay method to evaluate drug interaction effects.

### Apoptosis assay

4T1 cells were seeded in 6-well plates at a density of 2 × 10^5^ cells per well and incubated overnight. The cells were then treated with culture medium containing DOX, MND, ART, MND-ART, or MND-ART-GEL. The final concentrations of DOX and ART in the medium were 4 and 32 µg/mL, respectively. After 48 h of incubation, the cells were collected and washed with PBS. They were then stained with Annexin V-AF647 and 4′,6-diamidino-2-phenylindole (DAPI) (1:200 Annexin V, 2 µg/mL DAPI, diluted in 1× binding buffer) at 4 °C for 30 min. The cells were subsequently washed with PBS and analyzed via flow cytometry (LSRFortessa; BD, USA).

### Mitochondrial membrane potential assay

4T1 cells were seeded in 6-well plates at a density of 2 × 10^5^ cells per well and incubated overnight. The cells were then treated with culture medium containing DOX, MND, ART, MND-ART, or MND-ART-GEL. The final concentrations of DOX and ART were 4 and 32 µg/mL, respectively. After 48 h of incubation, the cells were collected and washed with PBS. They were then stained with JC-10 dye (15 µM, diluted in Cell Staining Buffer) at 37 °C for 30 min, washed with PBS, and analyzed via flow cytometry.

### Caspase-3 activity assay

4T1 cells were seeded in 6-well plates at a density of 2 × 10^5^ cells per well and incubated overnight. Subsequently, the cells were treated with culture medium containing DOX, MND, ART, MND-ART, or MND-ART-GEL. The final concentrations of DOX and ART were 2 and 16 µg/mL, respectively. After 24 h of incubation, the cells were collected, washed with PBS, and stained with Z-DEVD-AFC (2.5 µg/mL, diluted in Cell Staining Buffer) at 37 °C for 30 min. The cells were subsequently washed with PBS and analyzed via flow cytometry.

### Detection of Intracellular ROS

4T1 cells were seeded in 12-well plates at a density of 2 × 10^5^ cells per well and incubated overnight. The cells were then treated with culture medium containing DOX, MND, ART, MND-ART, or MND-ART-GEL. The final concentrations of DOX and ART were 2 and 16 µg/mL, respectively. After 16 h of incubation, the cells were collected, washed with PBS, and stained with 2′,7′-dichlorodihydrofluorescein diacetate (10 µM, diluted in serum-free medium) at 37 °C for 30 min. Thereafter, the cells were washed with PBS and analyzed via flow cytometry.

### Western blot analysis

4T1 cells were seeded in 6-well plates at a density of 3 × 10^5^ cells per well. After overnight incubation, the cells were treated with culture medium containing DOX, MND, ART, MND-ART, or MND-ART-GEL. The concentrations of DOX and ART were 240 ng/mL and 1.92 µg/mL, respectively. After 48 h of incubation, the cells were washed with PBS and lysed using RIPA lysis buffer supplemented with protease and phosphatase inhibitors. For tumor tissue samples, approximately 100 mg of tumor tissue was weighed and homogenized in 1 mL of RIPA buffer containing protease and phosphatase inhibitors using a precooled tissue homogenizer. The protein concentrations of both cell and tissue lysates were determined using a BCA protein assay kit. Equal amounts of protein were subjected to SDS-PAGE and transferred onto polyvinylidene fluoride membranes. The membranes were blocked with protein-free blocking buffer and incubated overnight at 4 °C with primary antibodies. After washing, the membranes were incubated with appropriate secondary antibodies for 1 h at room temperature. The signals were detected using the Amersham ImageQuant 800 imaging system (Cytiva, Sweden).

### Scratch assay

4T1 cells were seeded in 6-well plates at a density of 1 × 10^6^ cells per well and cultured overnight until they reached full confluence. A scratch was made on the well surface using a 200 µL pipette tip, and detached cells were removed by washing with PBS. The cells were then treated with culture medium containing DOX, MND, ART, MND-ART, or MND-ART-GEL. The final concentrations of DOX and ART were 2 and 16 µg/mL, respectively. Wound closure was monitored and photographed under a microscope at 0, 12, and 24 h.

### Matrigel invasion assay

4T1 cells were seeded in the upper chamber of a Transwell insert (8 μm pore size) at a density of 5 × 10^4^ cells per well in serum-free medium and starved overnight. After removing the medium, the cells were treated with serum-free medium containing DOX, MND, ART, MND-ART, or MND-ART-GEL. The final concentrations of DOX and ART were 1 and 8 µg/mL, respectively. After 48 h of incubation, the cells on the lower surface of the membrane were fixed with 4% paraformaldehyde and stained with 1% crystal violet. After washing with distilled water, the migrated cells were imaged under a microscope.

### Colony formation assay

4T1 cells were seeded in 6-well plates at a density of 2 × 10³ cells per well and cultured overnight. They were then treated with culture medium containing DOX, MND, ART, MND-ART, or MND-ART-GEL. The final concentrations of DOX and ART were 240 ng/mL and 1.92 µg/mL, respectively. The cells were cultured until visible colonies (> 50 cells per colony) were formed. The colonies were fixed with 4% paraformaldehyde and stained with 1% crystal violet, washed with distilled water, and air-dried. Images of each well were captured using a digital camera, and the number of colonies was counted.

### Analysis of immunogenic cell death (ICD)-related markers

4T1 cells were seeded in 6-well plates at a density of 2 × 10^5^ cells per well and cultured overnight. The cells were then treated with culture medium containing DOX, MND, ART, MND-ART, or MND-ART-GEL, with final concentrations of DOX and ART of 240 ng/mL and 1.92 µg/mL, respectively. After 48 h of incubation, the cells were harvested and washed with PBS. The expression levels of calreticulin (CRT), major histocompatibility complex class I (MHCI), and Fas cell surface death receptor (Fas) were analyzed via flow cytometry. In parallel, cell lysates were collected for western blot analysis of HMGB1 expression. After the different treatments, the cells were fixed with 4% paraformaldehyde and permeabilized with 0.3% Triton X-100, and blocked with 5% bovine serum albumin (BSA) for 60 min. The cells adhered to the coverslips were then incubated overnight at 4 °C in a humidified chamber with primary antibodies against CRT or HMGB1 (1:200 dilution). Next day, the cells were incubated with Alexa Fluor 488-conjugated secondary antibodies (1:400 dilution) for 1 h at room temperature, followed by counterstaining of the nuclei with Hoechst 33,342. After washing with PBS, the coverslips were mounted on glass slides with glycerol gelatin and imaged using a BX53 fluorescence microscope (Olympus, Japan).

### Bone marrow-derived dendritic cell (BMDC) maturation and phagocytosis assays under direct stimulation and 4T1 co-culture conditions

Bone marrow cells were harvested from the femur and tibia of BALB/c mice and resuspended in RPMI-1640 complete medium after lysing the red blood cells. The cells were cultured in the presence of 20 ng/mL granulocyte-macrophage colony-stimulating factor, and half of the medium was replaced every other day. On day 6, loosely adherent immature BMDCs were collected and seeded in 12-well plates at a density of 5 × 10^5^ cells/well. The cells were then treated with MSU (4 µg/mL), Mn²^+^ (1.5 µg/mL), or the MnUA combination. Lipopolysaccharide (LPS; 1 µg/mL) was used as the positive control. After 48 h of incubation, the cells were harvested, washed with PBS, and stained with anti-CD11c-APC, anti-CD86-PE, and anti-MHC II-PerCP/Cy5.5 antibodies (1:100 dilution) at 4 °C for 30 min. After washing, the cells were analyzed with flow cytometry to determine the proportion of mature BMDCs (MHC II^+^CD86^+^).

For the phagocytosis assay, 4T1 cells were pre-labeled with an FITC probe. Briefly, 4T1 cells in the logarithmic growth phase were collected and incubated with FITC dye for 20 min, followed by thorough washing to remove excess free dye and resuspension for subsequent use. The FITC-labeled 4T1 cells were then pretreated for 48 h with culture medium containing DOX, MND, ART, MND-ART, or MND-ART-GEL, in which the concentration of DOX was 240 ng/mL or the concentration of ART was 1.92 µg/mL. After treatment, the cells were collected and co-incubated with BMDCs at a 1:1 ratio for 12 h. Following co-incubation, the cells were harvested, washed with PBS, and stained with an anti-CD11c-APC antibody for 30 min at 4 °C in the dark. After washing with PBS, the proportion of BMDCs that phagocytosed FITC-labeled 4T1 cells (CD11c⁺FITC⁺ cells) was analyzed using flow cytometry.

For the co-culture assay, 4T1 cells were pretreated with DOX, MND, ART, MND-ART, or MND-ART-GEL for 48 h using DOX and ART concentrations of 240 ng/mL and 1.92 µg/mL, respectively. Treated 4T1 cells were then co-cultured with BMDCs; the group treated with LPS (1 µg/mL) served as the positive control. After 24 h of co-culture, the cells were collected, washed with PBS, and stained with anti-CD11c-APC, anti-CD86-PE, and anti-MHC II-PerCP/Cy5.5 antibodies (1:100 dilution) at 4 °C for 30 min. After washing, the samples were analyzed using flow cytometry to evaluate the maturation of BMDCs (MHC II^+^CD86^+^).

### Macrophage polarization assay

RAW264.7 cells were seeded in 12-well plates at a density of 1 × 10^6^ cells/well and incubated overnight. The cells were then treated with a serum-free medium containing MSU (4 µg/mL), Mn²^+^ (1.5 µg/mL), or a MnUA combination. A group treated with LPS (1 µg/mL) was used as the positive control. After 24 h of incubation, the cells were harvested, washed with PBS, and stained with an anti-CD86-APC antibody (1:100 dilution) at 4 °C for 30 min. After washing with PBS, the cells were analyzed via flow cytometry to determine the proportion of M1-polarized macrophages (CD86^+^).

### In vivo antitumor study using BALB/c mice

An orthotopic 4T1 breast cancer model was established using female BALB/c mice (6–8-weeks-old; weight, 18–22 g) purchased from the Guangdong Medical Laboratory Animal Center. The mice were housed under specific pathogen-free conditions at 25 ± 2 °C with a 12 h light/dark cycle and had ad libitum access to food and water. All animal procedures were approved by the Institutional Animal Care and Use Committee of the Guangzhou University of Chinese Medicine (Approval No. PZ24115). 4T1 cells (1 × 10^6^ cells in 100 µL PBS) were injected into the mammary fat pad of female BALB/c mice after washing with PBS to remove serum. Once the tumors reached a volume of approximately 80 mm³, the mice were randomly divided into six groups (*n* = 5 per group): PBS, DOX, MND, ART, MND-ART, and MND-ART-GEL. The Treatments were administered via intratumoral injection every other day for a total of eight doses. The administered doses were equivalent to 4 mg/kg of DOX or 32 mg/kg of ART. Tumor dimensions (length and width) and body weight were measured using a caliper every two days, and tumor volumes were calculated using the following formula:

$$\text{Tumor volume} (\mathrm{mm}^{3}) = \mathrm{length} \times \mathrm{width}^{2}/2.$$

At the end of the experiment, the mice were euthanized, and whole blood, heart, liver, spleen, lungs, kidneys, tumor-draining lymph nodes (TDLNs), and tumors were harvested for imaging and weighing. Major organs (the heart, liver, spleen, lungs, and kidneys) were fixed in 4% paraformaldehyde for histological analysis. Tumor tissues were divided into three portions for flow cytometry, western blotting, and paraffin embedding (for hematoxylin and eosin (H&E) and immunohistochemical staining). TDLNs were processed for flow cytometry analysis.

### Post-surgical tumor recurrence model and in situ treatment

To simulate postsurgical recurrence, incomplete tumor resection was performed when the tumor volume reached approximately 200 mm³. The Mice were anesthetized with isoflurane, and the surgical area was sterilized. An incision was made on one side of the tumor, and the skin was separated from the tumor using surgical scissors. The majority of the tumor was excised, leaving a residual tumor mass of approximately 20 mm³. The wound was sutured, and the mice were placed in a warming chamber for recovery. The mice were randomly divided into three groups (*n* = 5): PBS, DOX, and MND-ART-GEL (DOX: 4 mg/kg; ART: 32 mg/kg). Treatment was administered via in situ injection every other day for a total of five doses. Tumor size was measured using a digital caliper, and tumor volume was calculated using the formula provided in the “In Vivo Antitumor Study in BALB/c Mice” section.

### Flow cytometry of tumor and lymph node immune cells

At the end of treatment, tumors were excised and digested with tumor tissue digestion buffer at 37 °C for 1 h. The resulting tumor cell suspensions were passed through a 200-mesh nylon cell strainer. After lysing red blood cells, the single-cell suspension was blocked with 1% BSA at room temperature for 15 min. The cells were then divided into four parts for the analysis of tumor cells, DCs, macrophages, and T cells. The Lymph nodes were harvested and gently ground on a 200-mesh nylon cell strainer in PBS to obtain single-cell suspensions. After passing through the strainer, the cells were blocked with 1% BSA at room temperature for 15 min and divided into three parts for the detection of DCs, macrophages, and T cells. All samples were incubated with specific antibodies at 4 °C for 30 min in the dark, followed by flow cytometry analysis.

### Enzyme-linked immunosorbent assay for cytokines

Tumor tissue (100 mg) was weighed and homogenized in 0.9 mL of cold PBS containing protease inhibitors using a precooled tissue homogenizer. The tissue lysate was centrifuged to remove debris, and the supernatant was collected for further analyses. The levels of IL-6, TNF-α, and IFN-γ in the tumor homogenates were measured using commercially available ELISA kits (BioLegend, USA) according to the manufacturer’s instructions.

### Statistical analysis

Statistical analysis was performed using IBM SPSS Statistics 26, and graphs were generated with GraphPad Prism 10. For comparison between two groups, an independent-sample *t*-test was used, with Levene’s test applied to assess variance homogeneity. For comparisons among three or more groups, normality was tested using the Shapiro–Wilk test. Data with *P* > 0.05 were considered normally distributed and expressed as mean ± standard deviation (SD), shown in bar graphs. Data with *P* < 0.05 were considered non-normally distributed and presented as median (P25–P75), shown in box plots. Nonparametric tests were used for non-normal data: Mann–Whitney U test for two groups and Kruskal–Wallis H test for multiple groups.

For normally distributed data, one-way ANOVA was used. If variances were homogeneous (*P* > 0.05), Tukey’s test was applied for more than three groups, and Bonferroni correction for three or fewer groups with equal sample sizes. When sample sizes were unequal, Scheffé’s test was used. If variances were unequal (*P* < 0.05), Welch’s ANOVA followed by Dunnett T3 post hoc test was performed. Statistical significance was indicated as follows: ns (not significant), *P < 0.05 (significant), and **P < 0.01 (highly significant). Asterisks placed above the bars indicate comparisons versus the PBS group.

## Results and discussion

### Preparation and characterization of MND-ART-GEL

MnUA microparticles were first synthesized using a reverse microemulsion method. MSU and MnCl_2_ formed a MnUA microemulsion upon vigorous stirring, which was washed to remove the organic solvents and surfactants to obtain the MnUA particles. The MSU and Mn^2+^ contents in MnUA were quantified using a uricase digestion assay and ICP-MS, respectively, yielding an MSU content of 39.78% and a Mn^2+^ content of 7.63%.

**Fig. 1:**
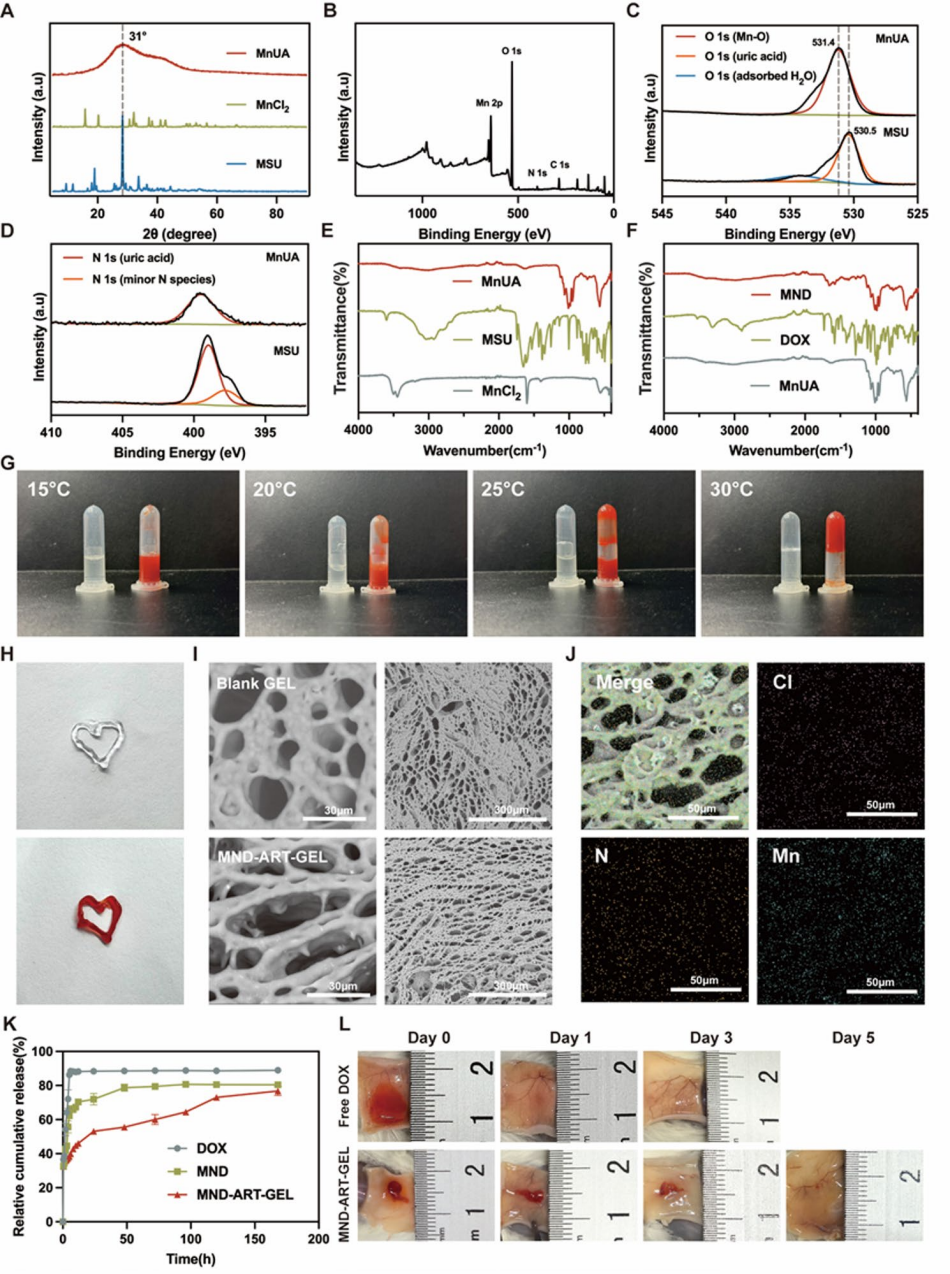
Structural characterization and physicochemical properties of MND-ART-GEL. (**A**) XRD patterns of MnUA, MnCl₂, and MSU. (**B**) XPS survey spectrum of MnUA. High-resolution O 1s (**C**) and N 1s (**D**) XPS spectra of MnUA and MSU. FTIR spectra of MnCl_2_, MSU, MnUA (**E**) and MnUA, DOX, MND (**F**). (**G**) Inversion test of blank GEL and MND-ART-GEL at different temperatures. (**H**) The injectability of blank GEL and MND-ART-GEL. (**I**) SEM images of blank GEL and MND-ART-GEL. (**J**) Elemental mapping of MND-ART-GEL. (**K**) Cumulative in vitro release profiles of DOX (*n* = 3). (**L**) In vivo retention and degradation of free DOX and MND-ART-GEL at the injection site

In the XRD patterns (Fig. 1A), MSU exhibits a series of sharp and well-defined diffraction peaks, indicating high crystallinity. In contrast, MnUA showed markedly broadened diffraction features with a broad diffuse peak centered at approximately 31°, suggesting a significant reduction in the long-range crystalline order. This change in the diffraction behavior indicates that the introduction of Mn^2+^ disrupts the original lattice arrangement of MSU, rendering MnUA a low-crystallinity or amorphous structure, which is consistent with the structural reorganization induced by metal–ligand coordination.

XPS analysis further supported the coordination structure of MnUA from the perspective of its chemical environment. The XPS survey spectrum of MnUA (Fig. 1B) clearly displays Mn 2p, O 1s, N 1s, and C 1s signals, confirming the successful incorporation of Mn into the system. In the high-resolution O 1s spectrum (Fig. 1C), the main O 1s peak of MnUA exhibited a positive shift compared with that of MSU, indicating the involvement of oxygen atoms in the interactions with Mn^2+^. The XPS survey spectrum (Fig. 1B, S1A) shows a pronounced decrease in the intensity of the N signal for MnUA relative to MSU, and the high-resolution N 1s spectrum (Fig. 1D) displays peak broadening, suggesting that the nitrogen-containing groups of the urate ligand participate in interactions with Mn^2+^ and experience reduced surface accessibility or coordination shielding. In the Mn 2p spectrum (Fig. S1B), MnUA predominantly exhibits the characteristic features of Mn^2+^ with no obvious signals corresponding to higher oxidation states, indicating that Mn remains in a stable divalent coordination state within MnUA.

The FTIR results were highly consistent with the conclusions drawn from the XRD and XPS results. FTIR spectra of MSU, MnCl_2_, and MnUA (Fig. 1E) revealed that the characteristic peaks of MSU at 1770–440 cm^−^¹—corresponding to C = O stretching of the ketone group and purine ring vibrations—were replaced by two new peaks at 1160–880 cm^−^¹ (C–O–Mn) and 560 cm^−^¹ (Mn–O) in MnUA. The enhanced 560 cm^−^¹ peak in MnUA compared to that in MnCl_2_ confirmed the formation of Mn–O coordination bonds. Additionally, the disappearance of the broad peak at 3200–2600 cm^−^¹ in MSU indicates that the amino group (–NH) on the purine ring may also coordinate with Mn²^+^, forming Mn–N bonds that stabilize the MnUA structure.

MnUA was then incubated with the DOX solution to form the drug-loaded MnUA-DOX (MND). The DOX content in the supernatant after incubation and centrifugation was quantified using HPLC, and the amount of loaded DOX was calculated accordingly. After drying and weighing the MND, the drug loading content and encapsulation efficiency were determined to be 35.45% and 97.50%, respectively. These results indicate that MND exhibits both a high drug -loading capacity and encapsulation efficiency, demonstrating that MnUA is an effective drug carrier. Notably, DOX loading could be achieved directly in an aqueous solution without the need for alkaline agents such as triethylamine to deprotonate DOX, as is commonly required in conventional loading strategies [32–35], highlighting the simplicity of the loading process.

The FTIR spectrum (Fig. 1F) indicated that drug loading occurred primarily via physical adsorption, as the characteristic peaks of DOX (e.g., –NH/–OH at 3530 and 3310 cm^−^¹, and anthraquinone ring vibrations between 1740 and 400 cm^−^¹) disappeared, whereas the MnUA skeleton peaks at 1010 and 560 cm^−^¹ remained unchanged, suggesting the physical adsorption of DOX rather than its chemical binding. Zeta potential measurements (Fig. S2) provided direct evidence for the formation mechanism of the MND. MnUA exhibited a pronounced negative surface potential in aqueous solution, arising from the negatively charged functional groups exposed on the urate anionic framework, whereas DOX molecules carry a positive charge under physiological conditions. Upon assembling DOX with MnUA to form MND, the zeta potential of the system shifted markedly toward neutrality, indicating effective electrostatic interactions between positively charged DOX and negatively charged MnUA. Collectively, the FTIR and zeta potential results demonstrated that MND construction was primarily driven by electrostatic adsorption between DOX and MnUA rather than by covalent modification or complex chemical reactions.

Pluronic F127 is a triblock copolymer consisting of a central hydrophobic polypropylene oxide block and two hydrophilic polyethylene oxide blocks that exhibit temperature-dependent self-assembly [36]. The blank hydrogel remained in the liquid form at temperatures between 15 and 25 °C but gelled at temperatures of ~ 30 °C (Fig. 1G). MND-ART-GEL retained this behavior, indicating its suitability for in situ gelation at physiological temperatures, which is ideal for local and postoperative tumor therapies. MND and ART were uniformly dispersed in a 22% Pluronic F127 thermosensitive hydrogel via ultrasonic mixing with 6% Pluronic film hydration, resulting in an injectable MND-ART-GEL (Fig. 1H). The drug loading content and encapsulation efficiency of ART in the hydrogel were 6.82% and 97.48%, respectively.

We systematically evaluated the viscoelastic properties of Blank GEL and MND-ART-GEL by measuring their storage modulus (G′) and loss modulus (G″). Temperature sweep (Fig. S3A) measurements showed that the G′ and G″ of MND-ART-GEL intersected at approximately 20°C, followed by a rapid increase in both moduli between 20 and 25°C, and reached a stable plateau near 30°C. These results indicate that MND-ART-GEL undergoes a sol–gel transition and forms a stable gel at temperatures close to 30°C. Frequency sweep tests (Fig. S3B) revealed that within the angular frequency range of 0.1–100 rad/s, G′ consistently remained higher than G″ for both hydrogels, indicating the typical solid-like behavior and good structural stability of the hydrogel networks. Strain sweep measurements (Fig. S3C) showed that G′ remained stable under low -strain conditions and gradually decreased at higher strains. Compared to Blank GEL, MND-ART-GEL maintained higher G′ values over a broader strain range, suggesting improved tolerance to mechanical deformation. Steady-shear viscosity measurements (Fig. S3D) demonstrated pronounced shear-thinning behavior for both hydrogels. As the shear rate increased from 0.1 to 100 1/s, the viscosity decreased markedly, indicating good injectability under the shear conditions. Step-strain tests were performed to assess the structural recovery behavior of the hydrogels (Fig. S3E). Under high strain, the G′ of both hydrogels rapidly decreased, whereas it quickly recovered upon returning to a low strain. This process was reproducible over multiple cycles with negligible mechanical degradation, indicating good self-recovery. Compared with Blank GEL, MND-ART-GEL exhibited comparable or slightly enhanced structural recovery capabilities. Overall, drug loading did not significantly compromise the G’ and G″ or shear-thinning behavior of the hydrogel. Moreover, both hydrogels exhibited good structural recovery in step-strain tests, indicating that the system maintained stable, injectable, and reproducible mechanical properties after drug loading. Scanning electron microscopy (SEM) images revealed a uniform porous gel network (~ 20 μm, Fig. 1I), and energy dispersive spectroscopy (EDS) mapping showed a homogeneous distribution of Mn, N, and Cl (Fig. 1J), indicating an even dispersion of MND within the gel.

Drug release profiles were evaluated using HPLC. As shown in Fig. 1K, ~ 90% of the free DOX was released within 6 h, and complete release was eventually achieved. In contrast, MND achieved a sustained -release plateau at ~ 80% over 48 h. MND-ART-GEL exhibited the slowest initial release and a sustained profile, with ~ 76% release achieved by day 7. As shown in Fig. S4, free ART was almost completely released within 2 h, whereas ART in the MND-ART-GEL formulation reached a plateau at 48 h, with approximately 70% of the drug being released. These results indicate that both MnUA particle encapsulation and hydrogel formulation confer sustained-release properties, enabling prolonged drug exposure and potentially reducing the systemic toxicity associated with conventional high-dose chemotherapies. In contrast, the relatively safe drug ART was released at a faster rate from the hydrogel, allowing for an early therapeutic effect. To evaluate the in vivo degradation or dissolution behavior, free DOX and MND-ART-GEL were subcutaneously injected into female BALB/c mice. At predetermined time points, the skin surrounding the injection sites was carefully excised to observe the retention and degradation characteristics of the MND-ART-GEL. As shown in Fig. 1L, a well-defined, uniformly red hydrogel was visible immediately after injection, whereas the free DOX appeared in a solution-like state. MND-ART-GEL formed a stable localized gel depot at the injection site, which gradually decreased in size over the following days and completely disappeared by day 5, indicating sustained and controllable local retention and release behavior. In contrast, free DOX diffused rapidly and was no longer detectable at the injection site within 1 day of injection. In addition, the biosafety of Blank GEL was evaluated in vitro and in vivo. Cytotoxicity assays performed on 4T1 cells demonstrated that Blank GEL treatment did not induce significant cytotoxicity (Fig. S5). Consistently, subcutaneous injection of Blank GEL into healthy mice showed good local tolerance. H&E staining of skin tissues at the injection sites (Fig. S6) revealed no obvious inflammatory infiltration or tissue damage, indicating the good biocompatibility of the hydrogel.

### Evaluation of the synergistic cytotoxicity of MND and ART against 4T1 cells

**Fig. 2:**
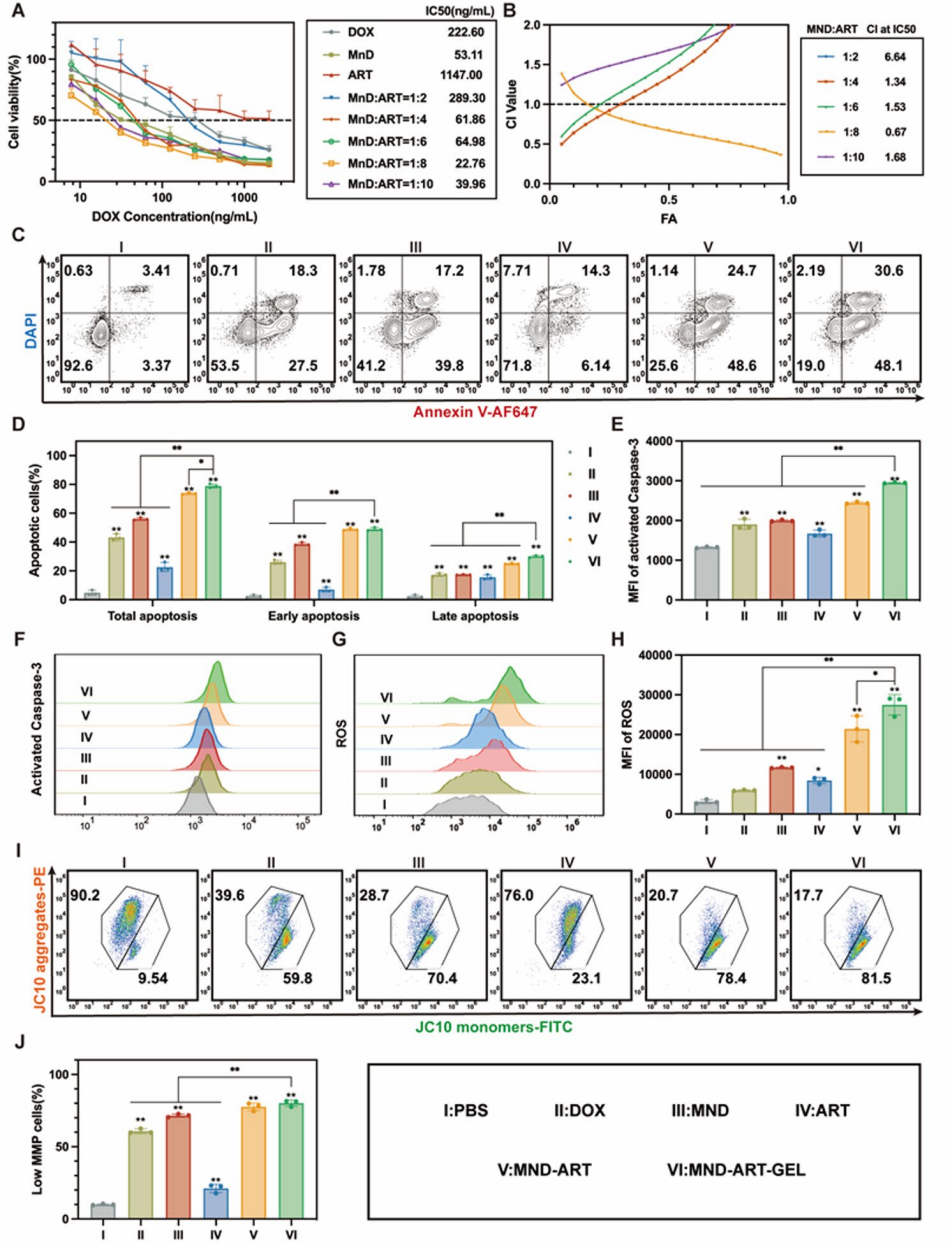
In Vitro Antitumor Efficacy Evaluation of MND-ART-GEL. (**A**) Cell viability curves and IC₅₀ values of 4T1 cells treated with different drug combinations (*n* = 3). (**B**) CI-FA plot and CI values at IC₅₀ for different drug combinations. Representative dot plots (**C**) and quantitative analysis (**D**) of Annexin V-DAPI staining to assess apoptosis in 4T1 cells (*n* = 3). Quantitative analysis (**E**) and representative histograms (**F**) of Caspase-3 activity in 4T1 cells (*n* = 3). Representative histograms (**G**) and quantitative analysis (**H**) of intracellular ROS levels in 4T1 cells (*n* = 3). Representative dot plots (**I**) and quantitative analysis (**J**) of JC-10 staining to evaluate mitochondrial membrane potential (MMP) in 4T1 cells (*n* = 3). Statistical annotations: *P < 0.05, **P < 0.01 vs. PBS group; ns, not significant

A CCK-8 assay was performed to determine the optimal drug ratio for combining MND and ART. As shown in Fig. 2A, DOX, a first-line chemotherapeutic agent, effectively inhibited the viability of 4T1 cells, whereas ART exerted a weaker cytotoxic effect, acting primarily as an adjuvant. Compared with DOX alone, MND exhibited a lower half-maximal inhibitory concentration (IC_50_), indicating the enhanced efficacy of DOX-loaded MnUA microparticles at reduced doses. When MND was combined with ART, the IC_50_ values at 1:8 and 1:10 ratios were further reduced, with the lowest value observed at the 1:8 ratio.

The combination index (CI), a quantitative metric for drug interaction, was used to evaluate synergism (CI < 1) or antagonism (CI > 1). As shown in Fig. 2B, the CI values at the 1:8 combination ratio were consistently less than 1 across most fractionally affected (FA) levels, with the lowest CI at IC_50_, indicating a favorable synergistic effect of combination therapy. Therefore, a 1:8 ratio was selected for the subsequent experiments.

### MND-ART-GEL induces apoptosis and ROS accumulation in 4T1 cells in vitro

Apoptosis and caspase-3 activation in 4T1 cells treated with various formulations were assessed using flow cytometry. All treatments induced apoptosis and caspase-3 activation at varying degrees. Compared with free DOX, the MND formulation and its combination with ART in the hydrogel form significantly increased the proportion of apoptotic cells, with MND-ART-GEL treatment resulting in the highest apoptosis rate and caspase-3 activation (Fig. 2C-F). These results indicate that, at equivalent drug dosages, the hydrogel formulation is more effective in inducing apoptosis in tumor cells.

DOX induces ROS accumulation, which damages tumor cell DNA and contributes to its cytotoxicity [37]. Moreover, ART contains an endoperoxide bridge that reacts with intracellular iron ions, leading to additional ROS production [38]. These ROS exacerbate DNA damage and mitochondrial dysfunction, indicating that ROS generation is one of the primary mechanisms underlying MND-ART-GEL–induced apoptosis.

To further confirm this hypothesis, intracellular ROS levels and mitochondrial membrane potential (MMP) were evaluated using flow cytometry. Compared to the DOX and MND groups, MND-ART-GEL significantly elevated ROS levels (Fig. 2G-H) and increased the proportion of cells with depolarized mitochondrial membranes (low MMP) (Fig. 2I-J). These findings confirm that MND-ART-GEL induces tumor cell death primarily through ROS-mediated mechanisms.

### MND-ART-GEL induces ICD and surface immunomarker exposure in 4T1 cells

**Fig. 3:**
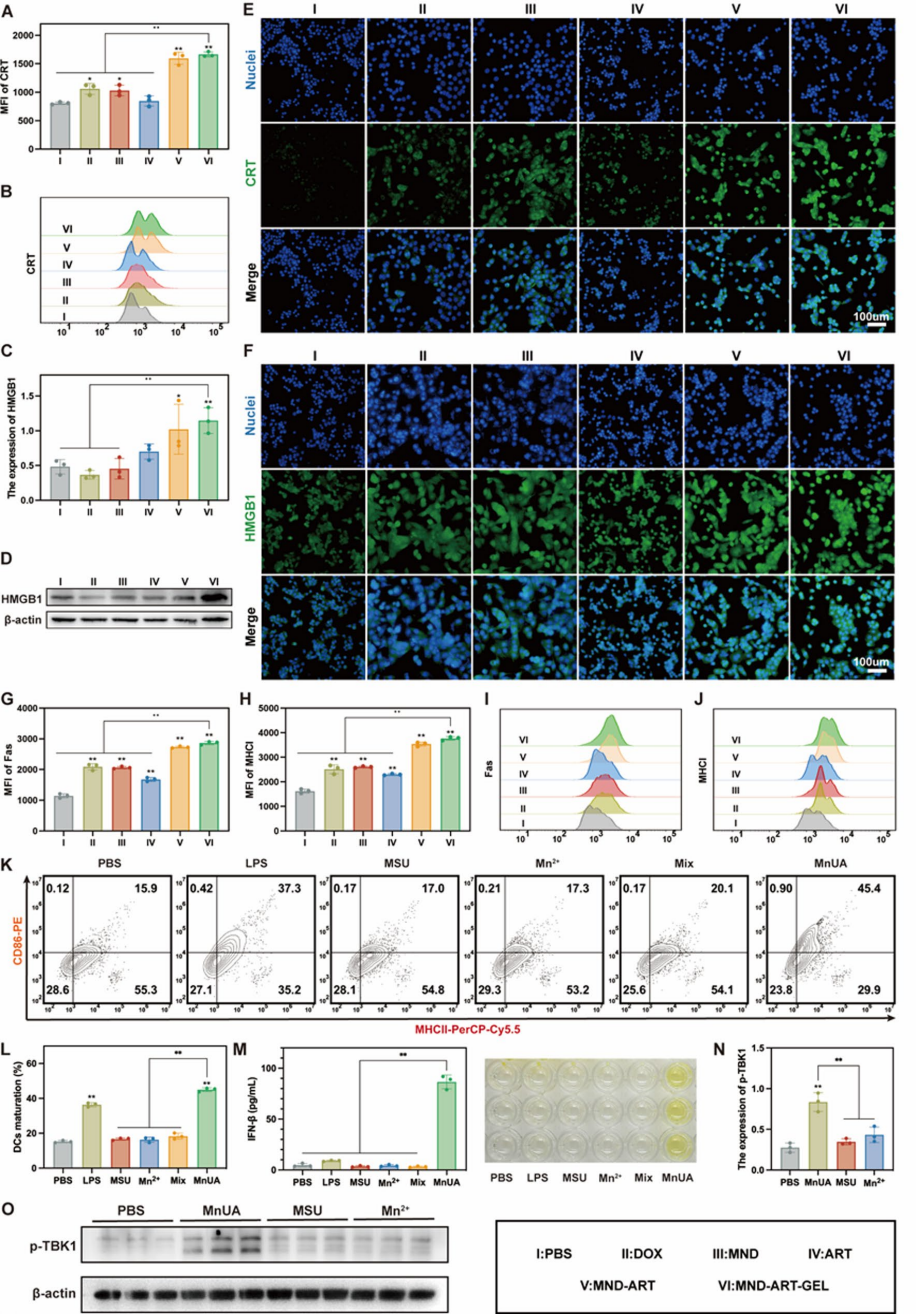
MND-ART-GEL Induces ICD and Promotes Immune Responses In Vitro. Quantitative analysis (**A**) and representative histogram (**B**) of CRT expression in 4T1 cells (*n* = 3). Semi-quantitative analysis (**C**) and western blot images (**D**) of HMGB1 expression in 4T1 cells (*n* = 3). Immunofluorescence images of CRT (**E**) and HMGB1 (**F**) expression in 4T1 cells. Quantitative analysis of Fas (**G**) and MHCI (**H**) expression in 4T1 cells. Representative flow cytometry histogram of Fas (**I**) and MHCI (**J**) expression in 4T1 cells. Representative flow cytometry scatter plots (**K**) and quantitative analysis (**L**) of BMDC maturation (CD86⁺MHCII⁺ cells%, *n* = 3). Quantification of IFN-β (**M**) in BMDCs culture supernatants (*n* = 3). Western blot analysis of p-TBK1 (**N**) protein expression in BMDCs (*n* = 3). (**O**) Western blot images of BMDCs with drugs treated. Statistical annotations: *P < 0.05, **P < 0.01 vs. PBS group; ns, not significant

DOX is a well-established ICD inducer that triggers endoplasmic reticulum (ER) stress in tumor cells, thereby promoting ICD [39]. As MND-ART-GEL elevates intracellular ROS levels, it may enhance ER stress-mediated ICD [40, 41]. The expression levels of CRT and high mobility group box 1 (HMGB1), two hallmark ICD-associated DAMPs, were evaluated to assess the ICD-inducing ability of the MND-ART-GEL. Among all treatment groups, MND-ART-GEL induced the highest expression levels of both CRT and HMGB1, significantly exceeding those observed in the DOX, MND, and ART groups (Fig. 3A-D). This was further validated by immunofluorescence imaging, wherein the 4T1 cells treated with MND-ART-GEL displayed the brightest green fluorescence signal (Fig. 3E, F).

Flow cytometry analysis of surface immune markers revealed that 4T1 cells treated with MND-ART-GEL exhibited increased expression of apoptosis-associated factor Fas and MHCI molecules (Fig. 3G–J). This enhancement in the expression of immunogenic markers indicated that MND-ART-GEL treatment rendered tumor cells more recognizable and susceptible to immune cell-mediated cytotoxicity.

### MND-ART-GEL activates immune cells in vitro

DCs are the most potent antigen-presenting cells (APCs). Mature DCs express high levels of MHC I/II and costimulatory molecules, such as CD80 and CD86, enabling them to present tumor antigens to T cells and initiate immune responses. Tumor-associated macrophages (TAMs) are a major component of the TME and exhibit two phenotypes: M1 and M2. M1-like TAMs exert antitumor activity by sustaining inflammation, recruiting immune cells, and phagocytosing tumor cells, whereas M2-like TAMs generally promote tumor progression.

To evaluate the immunostimulatory effects of MnUA, primary BMDCs and RAW264.7 macrophages from BALB/c mice were incubated with MnUA. Flow cytometry analysis revealed that MnUA treatment significantly increased the proportion of CD86^+^MHCII^+^ BMDCs (Fig. 3K-L), surpassing the effects observed in the MSU or Mn²^+^ single-agent groups and exceeding those in the free Mn^2+^ + MSU mixture group. These results indicate that MnUA does not act through a simple additive effect but exhibits emergent immunostimulatory properties beyond physical admixture. Similarly, the percentage of CD86^+^ RAW264.7 macrophages markedly increased following MnUA exposure (Fig. S7). These findings indicate that MnUA acts as a potent immune adjuvant capable of promoting DC maturation and polarizing TAMs toward the M1 phenotype.

To further elucidate the mechanism underlying MnUA-mediated immune activation, we examined the key components of the STING pathway. Western blotting and ELISA showed that MnUA treatment significantly increased the phosphorylation levels of STING and TBK1 (Fig. 3N, O, S8), accompanied by elevated IFN-β expression (Fig. 3M). Importantly, activation of the STING–TBK1–IFN-β axis was more pronounced in the MnUA group than in cells treated with equivalent concentrations of free Mn²⁺ and MSU alone.

Previous studies have demonstrated that MSU crystals activate NF-κB signaling [24]. In parallel, recent evidence indicates that NF-κB activation can prevent the intracellular degradation of activated STING, thereby amplifying STING-associated signaling cascades [23]. Based on these findings, together with our experimental observation of enhanced STING–TBK1–IFN-β activation in the MnUA group, we speculate that the superior immunostimulatory effect of MnUA may result from MSU-mediated NF-κB priming, which augments STING pathway activation in the presence of Mn²⁺.Collectively, these results suggest that MnUA may promote innate immune activation through the coordinated engagement of NF-κB and STING signaling pathways.

Given that ICD facilitates the release of tumor antigens and enhances the recognition and uptake of tumor cells by antigen-presenting cells, we further evaluated the effects of differently treated 4T1 cells on the phagocytic function of BMDCs. As shown in Fig. S9, flow cytometric analysis revealed a relatively low proportion of CD11c⁺FITC⁺ double-positive cells in the PBS group, indicating the limited basal phagocytic capacity of BMDCs for tumor cells. In contrast, the MND-ART-GEL group exhibited the highest proportion of CD11c⁺FITC⁺ cells, and quantitative analysis demonstrated that the proportion of BMDCs that phagocytosed 4T1 cells was significantly higher in the MND-ART-GEL group than in the PBS- and DOX-treated groups (**P < 0.01). These results indicate that MND-ART-GEL more effectively promotes the phagocytosis of tumor cells by BMDCs, and this enhanced antigen uptake capacity provides an important foundation for subsequent dendritic cell maturation and activation of antitumor immune responses.

After confirming that drug-treated 4T1 cells could be efficiently internalized by BMDCs, we investigated whether this process was accompanied by alterations in the functional status of BMDCs. Subsequently, the expression of BMDC maturation-associated phenotypic markers was examined in the co-culture system to evaluate the effects of tumor cell-mediated immune activation. As shown in Fig. S10, compared with the blank control, BMDCs co-cultured with PBS-treated 4T1 cells exhibited marked immunosuppression, with reduced maturation, likely due to the suppressive cytokines or metabolites secreted by tumor cells [42, 43]. In contrast, the MND-ART-GEL–treated group exhibited the highest DC maturation, significantly exceeding that of the other groups, indicating that ICD triggered by MND-ART-GEL could reverse the immunosuppressive TME.

### MND-ART-GEL suppresses JAK2-STAT3 and inhibits invasion and metastasis of 4T1 cells

In TNBC, the STAT3 signaling pathway is frequently hyperactivated, promoting tumor proliferation, invasion, metastasis, and immune evasion, while contributing to chemoresistance [25, 26]. ART inhibits STAT3 by preventing its phosphorylation and nuclear translocation, thereby reducing its DNA-binding ability [27]. This mechanism may be a key contributor to the antitumor activity of the MND-ART-GEL system.

**Fig. 4:**
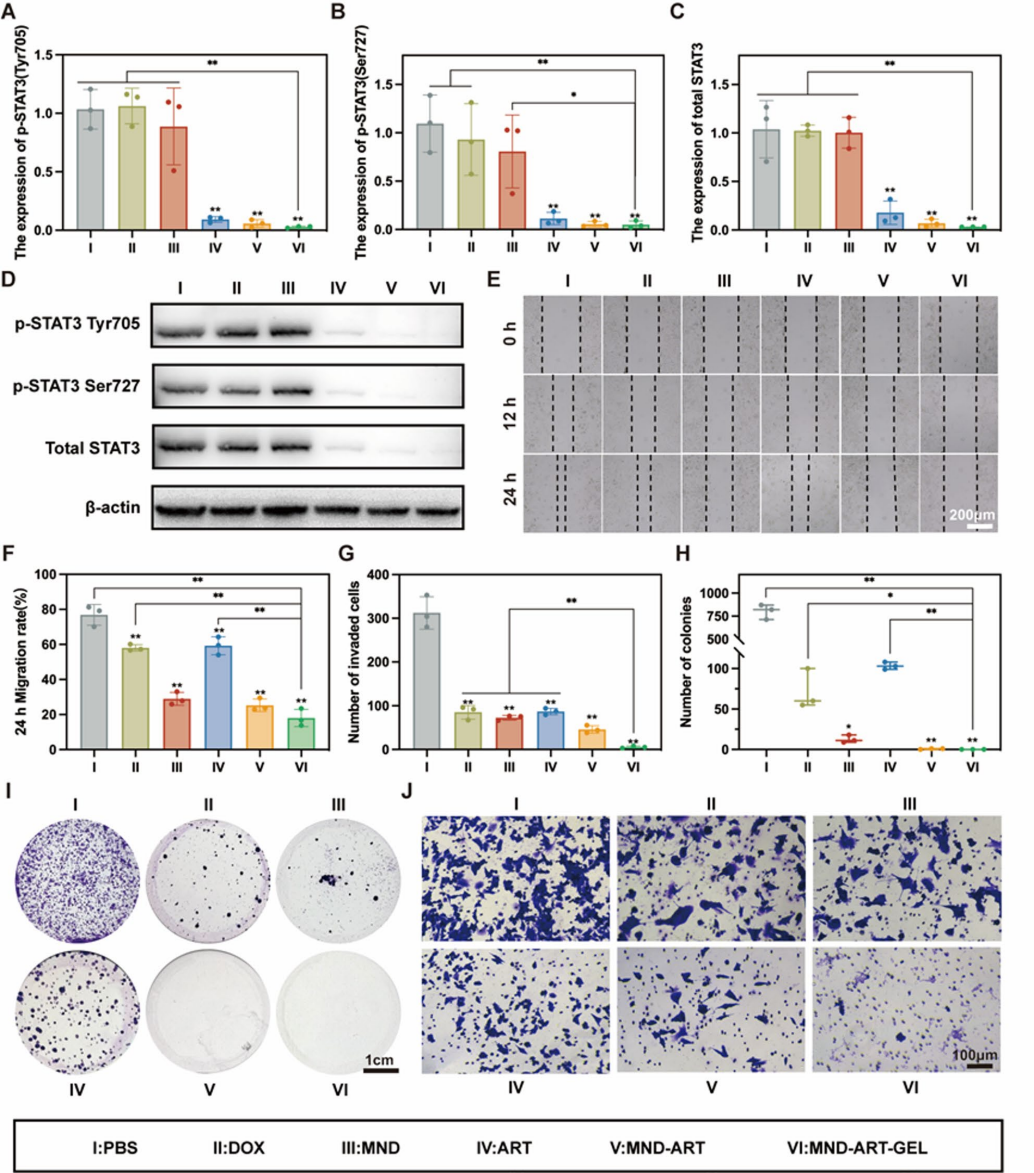
MND-ART-GEL Inhibits 4T1 Cell total STAT3 and p-STAT3 levels, Invasion and Migration by Downregulating STAT3. Western blot analysis of p-STAT3 (Tyr705 (**A**) and Ser727 (**B**)) and total STAT3 (**C**) protein expression in 4T1 cells (*n* = 3). (**D**) Western blot images of 4T1 cells with drugs treated. (**E**) Bright-field images of wound healing assay at 12 h and 24 h after treatment. (**F**) Quantification of migration rate at 24 h (*n* = 3). (**G**) Quantification of invaded cells (*n* = 3). (**H**) Quantification of colony numbers (*n* = 3). (**I**) Images of colony formation assay. (**J**) Bright-field images of Transwell invasion assay. Statistical annotations: *P < 0.05, **P < 0.01 vs. PBS group; ns, not significant

Western blotting was used to detect total STAT3 and phosphorylated STAT3 (Tyr705 and Ser727) levels in the treated 4T1 cells. As shown in Fig. 4A–D, the drugs containing ART (free ART, MND-ART, and MND-ART-GEL) significantly downregulated the levels of both p-STAT3 and total STAT3. To further investigate the mechanism underlying STAT3 downregulation, we examined the protein expression levels of its upstream signaling molecule, JAK2, and phosphorylated JAK2 (p-JAK2). Western blot analysis demonstrated that MND-ART-GEL treatment markedly reduced p-JAK2 expression (Fig. S11), whereas total JAK2 levels remained relatively stable, indicating suppression of JAK2 activation. These results suggest that the inhibition of the JAK2-STAT3 signaling axis occurs at the phosphorylation level. Scratch, colony formation, and Transwell invasion assays further revealed that MND-ART-GEL effectively inhibited the migration, invasion, proliferation, and clonogenic potential of 4T1 cells (Fig. 4E–J), indicating its strong potential to suppress TNBC metastasis.

### Transcriptomic analysis of 4T1 cells treated with MND-ART-GEL

To elucidate the molecular mechanisms underlying the antitumor effects of MND-ART-GEL, transcriptome sequencing and differential gene expressssion analyses were performed on 4T1 cells treated with different formulations. As evident from the principal component analysis (PCA) and correlation matrix results (Fig. S12), samples within the same group exhibited high consistency, with clear intergroup differences, justifying the downstream analysis.

**Fig. 5:**
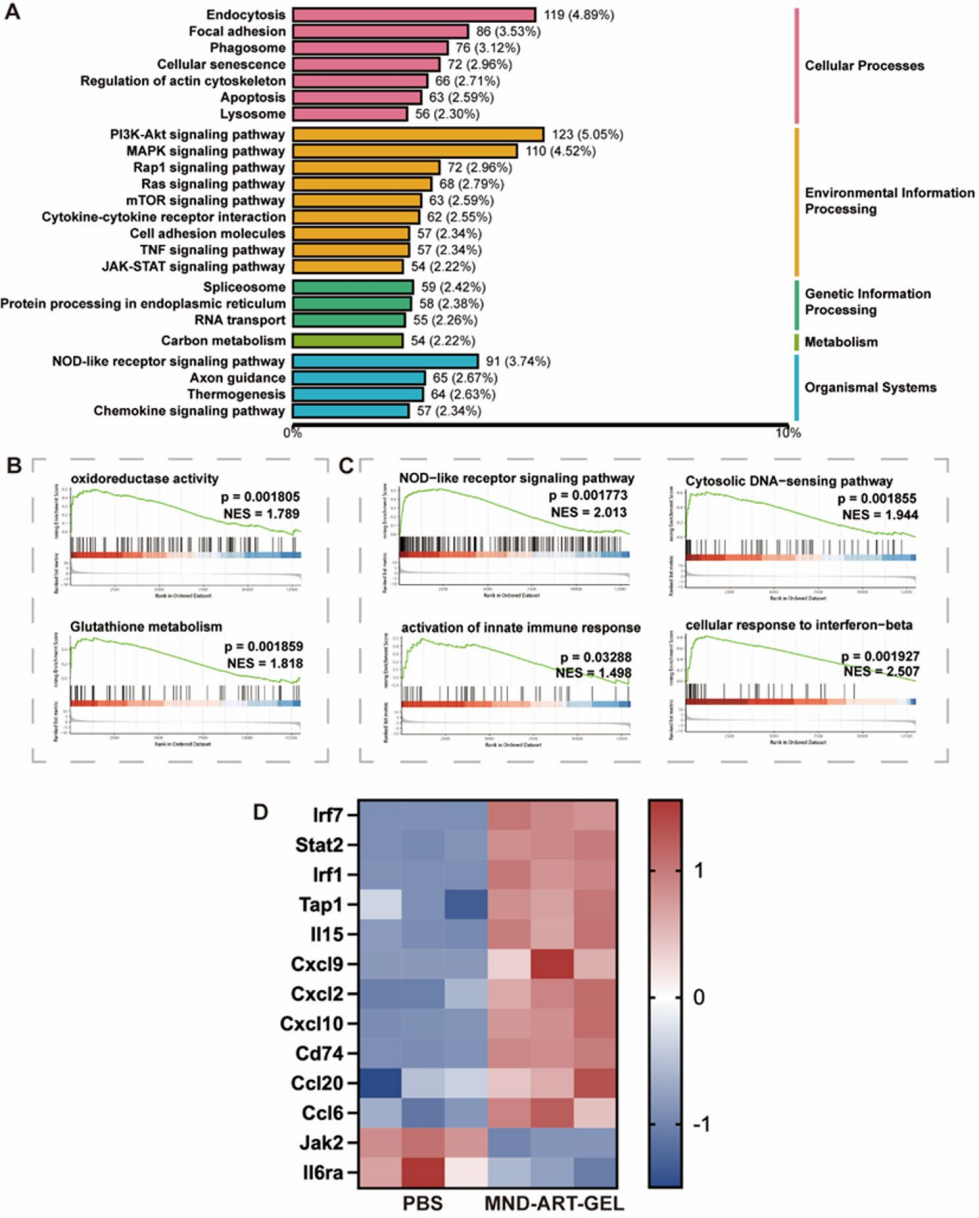
Transcriptomic Analysis of 4T1 Cells Treated with MND-ART-GEL (*n* = 3). (**A**) KEGG pathway classification of differentially expressed genes between PBS and MND-ART-GEL groups. (**B**) GSEA of selected pathways related to oxidative stress. (**C**) GSEA of selected pathways related to innate immune responses. (**D**) Heatmap of representative immune-related differentially expressed genes. Data are shown as row-scaled Z-scores

Gene ontology (GO) classification analysis revealed that differentially expressed genes were predominantly associated with immune system processes, responses to stimuli, and metabolic regulation at the biological process level, while intracellular and protein-containing complexes were enriched at the cellular component level, and binding- and catalytic-related functions were dominant at the molecular function level (Fig. S13). Consistently, the Kyoto encyclopedia of genes and genomes (KEGG) pathway classification further indicated that immune-related pathways and signal transduction processes constituted the major functional categories affected by MND-ART-GEL treatment (Fig. 5A), suggesting broad transcriptional reprogramming toward immune- and stress-associated states.

To move beyond descriptive pathway enrichment and identify mechanistically relevant signaling programs, a focused gene set enrichment analysis (GSEA) was performed, emphasizing pathways associated with oxidative stress and innate immune activation. This analysis revealed significant enrichment of oxidoreductase activity and glutathione metabolism, indicating the disruption of redox homeostasis and enhanced oxidative stress in MND-ART-GEL–treated cells (Fig. 5B). These transcriptomic changes are consistent with the pronounced intracellular ROS accumulation observed experimentally, supporting a redox imbalance–driven cellular stress response.

In parallel, multiple innate immune–related pathways were significantly enriched, including NOD-like receptor signaling, cytosolic nucleic acid sensing, activation of the innate immune response, and cellular response to interferon-beta (Fig. 5C). The coordinated enrichment of these pathways suggests that MND-ART-GEL–induced cellular stress and immunogenic perturbations effectively engage innate immune sensing mechanisms and type I interferon–associated transcriptional programs, providing a mechanistic basis for the observed immunogenic cell death and enhanced immune activation.

To further substantiate the pathway-level findings, a heatmap of representative immune-related differentially expressed genes was generated (Fig. 5D). Heatmap visualization demonstrated significant upregulation of interferon-associated transcription factors, including Irf7, Stat2, and Irf1, indicating activation of the type I interferon axis. Consistently, multiple chemokines involved in immune cell recruitment, such as Cxcl9, Cxcl10, Cxcl2, Ccl20, and Ccl6, were substantially elevated in the MND-ART-GEL group, suggesting enhanced immune infiltration potential. In parallel, antigen presentation–related genes, including Cd74 and Tap1, were upregulated, supporting improved antigen processing capacity. Notably, the key components of the IL-6/JAK signaling pathway, including Jak2 and Il6ra, were significantly downregulated, which aligned with the observed suppression of JAK2/STAT3 signaling at the protein level. Collectively, these transcriptional changes indicate that MND-ART-GEL simultaneously promotes interferon-driven immune activation while attenuating pro-tumorigenic JAK–STAT signaling.

Simultaneously, we further examined the transcriptomic data and found that, apart from the significant downregulation of several individual genes such as Jak2 and Il6ra, the JAK–STAT pathway was not significantly enriched at the transcriptional level. This suggests that the downregulation of STAT3 is more likely attributable to the oxidative stress-mediated inhibition of JAK2/STAT3 phosphorylation rather than transcriptional suppression [44, 45].

### In vivo antitumor efficacy and immune activation of MND-ART-GEL in an orthotopic 4T1 breast cancer model

**Fig. 6:**
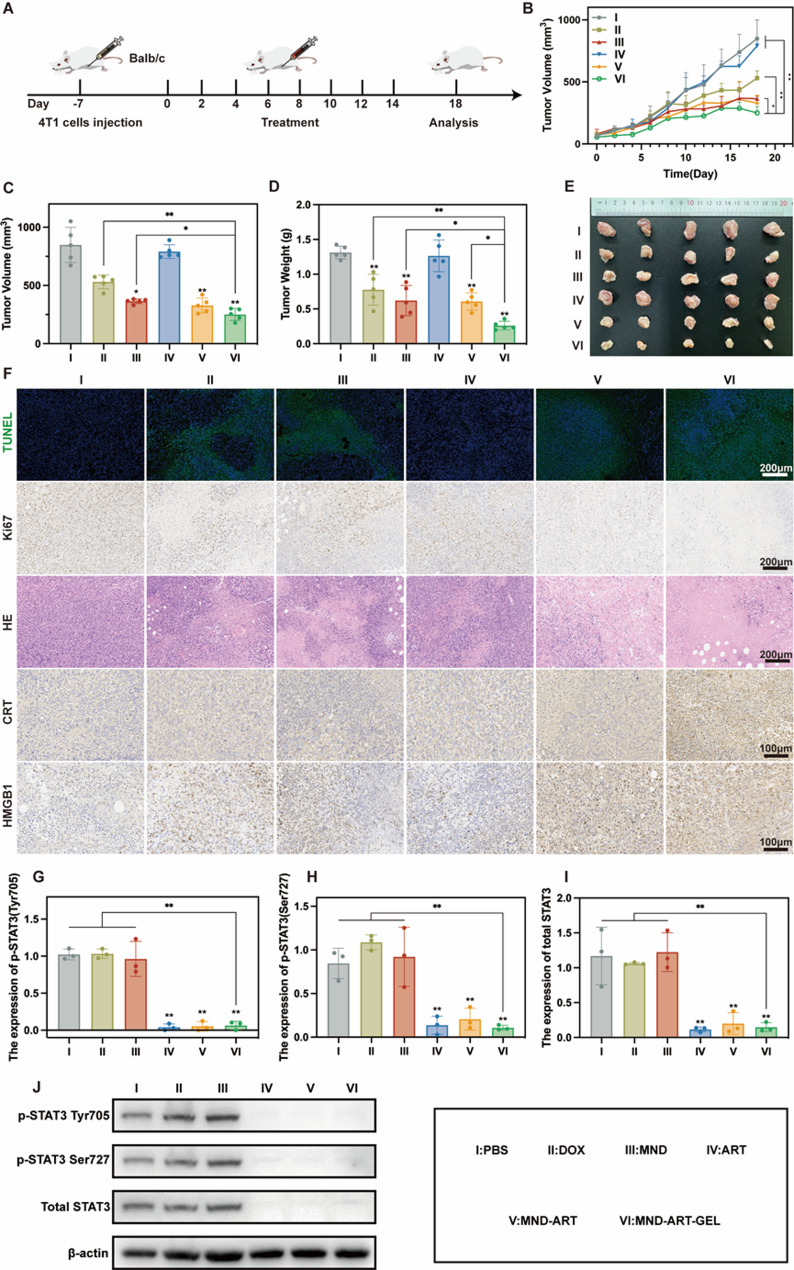
In Vivo Antitumor Efficacy of MND-ART-GEL in an Orthotopic 4T1 Breast Cancer Model. (**A**) Schematic diagram of tumor model establishment and treatment regimen. (**B**) Tumor growth curves in each treatment group (*n* = 5). Tumor volume (**C**) and weight (**D**) post-treatment (*n* = 5). (**E**) Representative tumor images after treatment. (**F**) TUNEL, Ki67, H&E, CRT, and HMGB1 staining of tumor sections. Western blot analysis of p-STAT3 (Tyr705 (**G**) and Ser727 (**H**)) and total STAT3 (**I**) expression in tumor tissues from different groups (*n* = 3). (**J**) Western blot images of tumor tissues in difference drugs treated groups. Statistical annotations: *P < 0.05, **P < 0.01 vs. PBS group; ns, not significant

Given the potent in vitro antitumor and immunostimulatory effects of MND-ART-GEL, its in vivo efficacy was evaluated using an orthotopic 4T1 breast tumor model in BALB/c mice. As shown in Fig. 6A, mice received intratumoral injections of different formulations (DOX 4 mg/kg or ART 32 mg/kg) every other day for a total of seven treatments. The ART group showed minimal tumor inhibition, whereas the DOX, MND, MND-ART, and MND-ART-GEL groups significantly suppressed tumor growth, with MND-ART-GEL exhibiting the greatest tumor inhibition, as evidenced by the lowest tumor weight and volume (Fig. 6B–E).

Histological analyses, including H&E, TUNEL, and Ki67 staining, were performed to assess tumor tissue morphology, apoptosis, and proliferation. H&E staining revealed only minor nuclear condensation in the ART group, whereas the DOX and MND groups showed increased areas of nuclear condensation, with MND also displaying nuclear dissolution, indicative of apoptosis and necrosis. Notably, MND-ART and MND-ART-GEL treatments led to extensive nuclear condensation, fragmentation, and dissolution, with the MND-ART-GEL group exhibiting near-complete nuclear degradation (Fig. 6F). TUNEL and Ki67 staining results corroborated these findings, demonstrating the highest levels of apoptosis and inhibition of proliferation in the MND-ART-GEL group.

To validate the in vitro findings of ICD induction and STAT3 suppression, HMGB1 and CRT expression was examined via immunohistochemistry, whereas STAT3 and phosphorylated STAT3 levels were analyzed via western blotting. As shown in Fig. 6F, HMGB1 release and CRT membrane translocation were significantly increased in the MND-ART and MND-ART-GEL groups compared to those in the PBS, DOX, MND, and ART control groups. Furthermore, STAT3 and its phosphorylated forms (Tyr705 and Ser727) were significantly downregulated in the ART-containing groups, consistent with the in vitro data (Fig. 6G–J). These results confirmed that MND-ART-GEL induced strong ICD and inhibited STAT3 signaling in vivo.

**Fig. 7:**
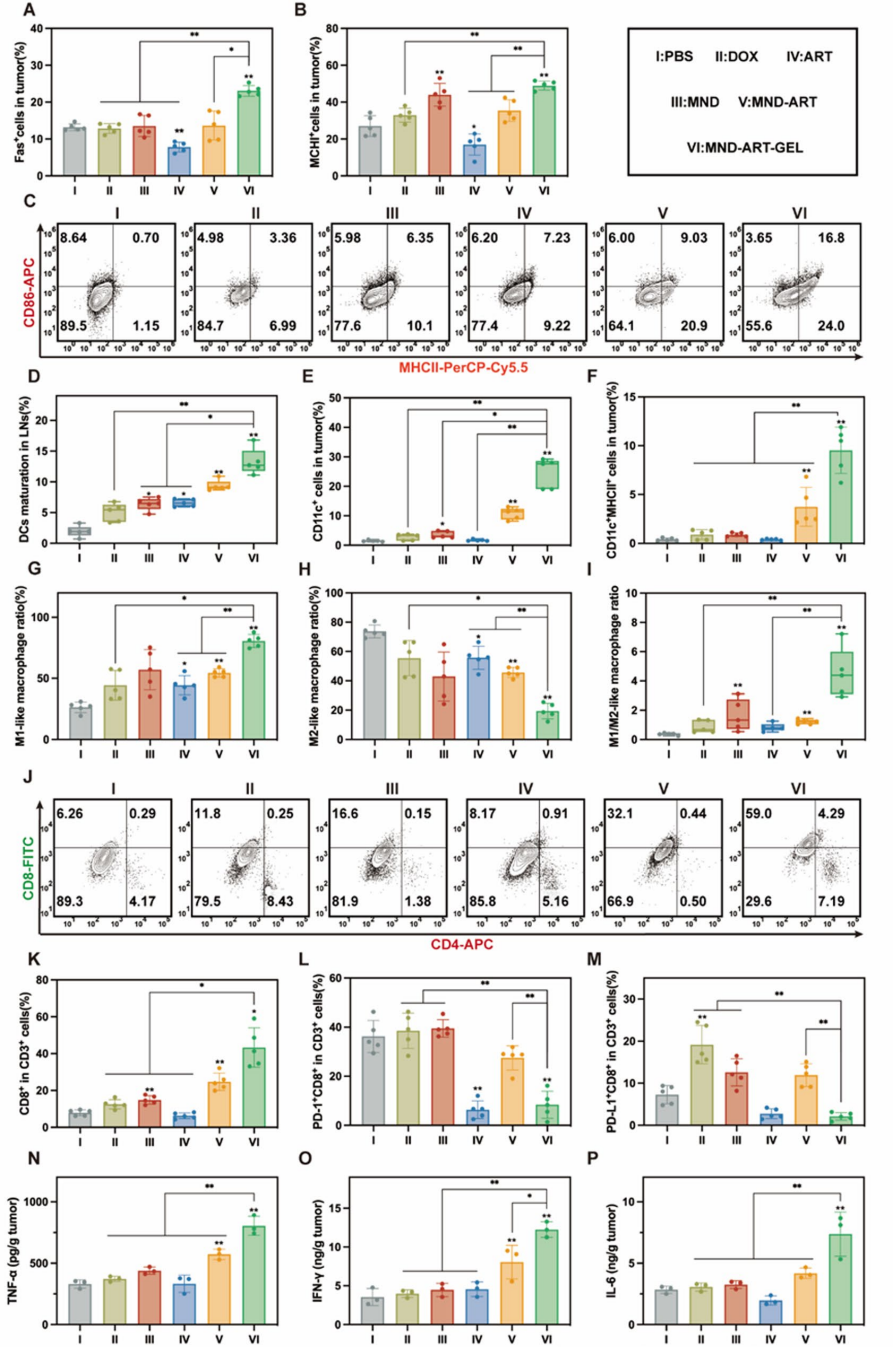
In Vivo Immune Profiling in the Orthotopic 4T1 Breast Cancer Model. Percentages of Fas⁺ (**A**) and MHCI⁺ (**B**) in tumor cells post-treatment (*n* = 5). Maturation of DCs in TDLNs (CD86⁺MHCII⁺ cells%, *n* = 5): representative plots (**C**) and quantification (**D**). Tumor-infiltrating DCs (CD11c⁺ cells%, *n* = 5) (**E**) and mature DCs (CD11c⁺MHCII⁺ cells%, *n* = 5) (**F**). Percentages of M1 macrophages (CD86⁺CD206⁻) (**G**), M2 TAMs (CD86⁺CD206⁺ and CD86⁻CD206⁺) (**H**), and M1/M2 ratio in tumors (*n* = 5) (**I**). Representative flow cytometry plots (**J**) and quantification (**K**) of CD8⁺ T cells (CD3⁺CD8⁺%, *n* = 5) in tumors post-treatment. CD8⁺PD-1⁺ (**L**), and CD8⁺PD-L1⁺ T cells% (**M**) in tumors (*n* = 5). Quantification of TNF-α (**N**), IFN-γ (O), and IL-6 (**P**) in tumor tissues (*n* = 3). Statistical annotations: *P < 0.05, **P < 0.01 vs. PBS group; ns, not significant

To further evaluate the immunomodulatory effects of MND-ART-GEL, flow cytometry was performed to analyze the immune cell populations in tumors and TDLNs (Fig. S14). The Surface expression of MHCI and Fas on tumor cells was first examined. Compared to the PBS group, the MND-ART-GEL group showed a two-fold increase in the percentage of Fas^+^ cells and a 1.8-fold increase in the percentage of MHCI^+^ cells, both of which were significantly higher than the respective percentages in the DOX and ART groups (Fig. 7A-B). These results indicate that MND-ART-GEL effectively enhances tumor cell immunogenicity in vivo, facilitating immune recognition and cytotoxicity.

Based on our findings that MnUA functions as an immune adjuvant and that MND-ART-GEL induced robust ICD, we evaluated DC activation in TDLNs and tumor tissue. The MND-ART-GEL group exhibited the highest proportion of mature DCs (CD86^+^MHCII^+^) in TDLNs, approximately 6.87-fold higher than that in the PBS group and significantly higher than that in the MND and ART groups (Fig. 7C-D). Although DC infiltration in tumors was relatively low, MND-ART-GEL treatment increased the abundance of CD11c^+^ DCs from 1.4% to 27.6% (Fig. 7E), providing a favorable immune environment. A similar trend was observed for MHCII^+^ DCs, the abundance of which increased from 0.4% in the PBS group to approximately 9.5% in the MND-ART-GEL group (Fig. 7F).

TAM profiling revealed that MND-ART-GEL significantly promoted M1 polarization and suppressed M2 polarization compared with other treatments (Fig. 7G-H). The M1/M2 ratio in tumors treated with MND-ART-GEL was approximately 4.5, which was 12.5-times higher than that in the PBS group (Fig. 7I).

We next assessed the percentage of CD8^+^ T cells, which are crucial for tumor cell killing and correlate with a favorable prognosis. MND-ART-GEL induced the highest CD8^+^ T-cell infiltration, which reached 43.3%, approximately 5.5-times that of the PBS group (Fig. 7J-K). Notably, MND-ART-GEL also reduced the expression of PD-1 and PD-L1 in CD8^+^ T cells (Fig. 7L, M). This finding indicates that MND-ART-GEL not only promotes T-cell infiltration but also alleviates T-cell exhaustion and immune suppression, thereby restoring cytotoxic activity. In addition, the ART-alone group exhibited reduced PD-L1 and PD-1 expression levels. Previous studies have reported that inhibiting the phosphorylation of STAT3 can reduce the expression of PD-L1 in tumor cells [46]; therefore, the observed decrease may be partially associated with ART-mediated STAT3 inhibition.

Finally, proinflammatory cytokines, including TNF-α, IFN-γ, and IL-6, were quantified in the tumor tissues using ELISA. These cytokines play essential roles in antitumor immunity by activating cytotoxic T cells, enhancing antibody production, and promoting immune cell recruitment to the TME [47–49]. The MND-ART-GEL group exhibited the highest levels of TNF-α, IFN-γ, and IL-6 among all groups (Fig. 7N–P), indicating a favorable shift in the immune microenvironment.

In summary, MND-ART-GEL effectively triggered ICD and downregulated STAT3, thereby remodeling the immunosuppressive microenvironment of TNBC and eliciting a robust antitumor immune response in vivo.

### Evaluation of the anti-recurrence efficacy of MND-ART-GEL after surgery

**Fig. 8:**
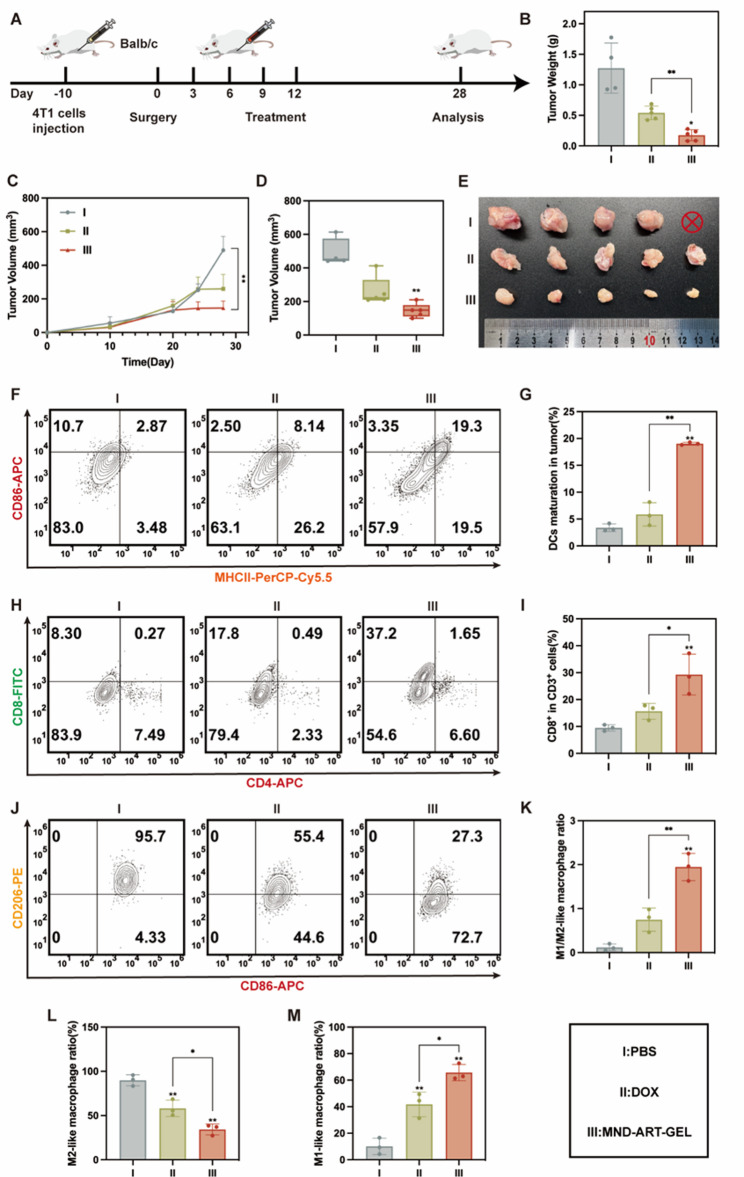
Postoperative Anti-Recurrence Efficacy and Immunological Evaluation of MND-ART-GEL. (**A**) Schematic diagram of the 4T1 postsurgical tumor recurrence model and treatment plan. Tumor weight (**B**), growth curves (**C**), and volume (**D**) in each group (*n* = 5, PBS group *n* = 4). (**E**) Representative tumor images post-treatment. DC maturation in tumor (CD86⁺MHCII⁺ cells%, *n* = 3): representative plots (**F**) and quantification (**G**). CD8⁺ T cell infiltration in tumors (CD3⁺CD8⁺ cells%, *n* = 3): representative plots (**H**) and quantification (**I**). Representative flow plots of macrophage subpopulations in tumor (**J**), percentages of M1/M2 ratio (**K**), M2 TAMs (CD86⁺CD206⁺ and CD86⁻CD206⁺) (**L**), and M1 macrophages (CD86⁺CD206⁻) (**M**) in tumors (*n* = 3). Statistical annotations: *P < 0.05, **P < 0.01 vs. PBS group; ns, not significant

Surgical resection remains the primary curative treatment for TNBC; however, the risk of postoperative recurrence is high and often detrimental to patient outcomes. To assess whether MND-ART-GEL can prevent tumor relapse after surgery, a postsurgical recurrence model was established. As illustrated in Fig. 8A, mice were randomly assigned to three groups (PBS, DOX, and MND-ART-GEL; DOX: 4 mg/kg; ART: 32 mg/kg; *n* = 5) and received five intratumoral injections every other day after the surgery. One mouse in the PBS group died of relapse before the study endpoint, and the remaining mice in the PBS group exhibited severe tumor regrowth. In contrast, MND-ART-GEL treatment effectively suppressed the growth of residual tumor cells, resulting in significantly reduced tumor weights and volumes compared with those in the PBS and DOX groups (Fig. 8B-E), indicating its robust anti-recurrence capability. Consistent with the tumor growth suppression observed in the recurrence model, Kaplan–Meier survival analysis (Fig. S15, *n* = 10 per group) was performed because recurrence is clinically associated with survival outcomes. MND-ART-GEL treatment significantly prolonged survival compared with that in the PBS and DOX groups. Specifically, the median survival time was 33.5 days in the PBS group and 42 days in the DOX group, whereas no mortality was observed in the MND-ART-GEL group within the 42-day observation period, indicating a marked survival benefit conferred by the hydrogel formulation.

Postoperative tumors typically present an immunosuppressive microenvironment. Therefore, flow cytometry was used to assess the immune cell profiles of relapsed tumors. MND-ART-GEL markedly enhanced DC maturation (Fig. 8F, G), CD8^+^ T-cell infiltration (Fig. 8H, I), and M1 macrophage polarization, while reducing the abundance of M2-type TAMs (Fig. 8J–M). The M1/M2 ratio in the MND-ART-GEL group was 16.25-times of that the PBS group (Fig. 8K). These results indicate that MND-ART-GEL successfully reversed the immunosuppressive state of postoperative residual tumors and reactivated antitumor immunity.

### MND-ART-GEL in vitro and in vivo safety evaluation

To further evaluate the safety profile of the formulation, cardiotoxicity was first assessed in vitro using HL-1 cardiomyocytes, given the well-recognized cardiac toxicity of doxorubicin. As shown in Fig. S16, DOX treatment markedly reduced HL-1 cell viability, whereas MND-ART-GEL exhibited significantly attenuated cytotoxicity, with increased cell viability and higher IC₅₀ values compared with free DOX. These results suggest that the incorporating DOX into the MnUA hydrogel system mitigates its direct cardiotoxic effects at the cellular level.

Representative H&E-stained lung sections (Fig. S17) were examined to evaluate pulmonary histopathological changes in the different treatment groups. In the PBS group, the lung tissues exhibited marked consolidation, characterized by reduced or collapsed alveolar spaces and prominent inflammatory cell infiltration. These changes may be associated with tumor-induced systemic inflammatory responses or pre-metastatic niche formation [50]. In contrast, mice treated with MND-ART-GEL displayed relatively preserved alveolar architecture with significantly alleviated inflammatory infiltration, indicating improved pulmonary histological status.

Throughout the treatment period, the body weights of the mice were monitored every two days. At the study endpoint, the major organs were harvested, weighed, and subjected to H&E staining. No significant differences in body weight or organ coefficients were observed between the groups (Fig. S18A, B). Although splenomegaly was noted—likely attributable to tumor-associated immune activation—it was not considered treatment-related toxicity. Histological examination revealed no evident pathological abnormalities in the major organs (Fig. S18C), supporting the overall biosafety of the formulation.

To further assess systemic safety, blood biochemical parameters were analyzed in healthy mice following subcutaneous injection (Fig. S19). No significant abnormalities were observed in the liver or renal function–related indicators, indicating that the formulation did not induce detectable systemic toxicity under the administered conditions.

Regarding manganese safety, it should be noted that the Mn^2+^ content in the formulation is relatively low and predominantly exists in a coordinated state within MnUA rather than as free ions. Combined with localized hydrogel delivery and the absence of observable systemic toxicity in vivo, these findings suggest that the administered dose falls within a tolerable safety range.

### Translational prospects for postoperative anti-recurrence therapy

In the current therapeutic landscape of TNBC, conventional chemotherapy remains the mainstay treatment but is often limited by systemic toxicity and tumor recurrence. Immune checkpoint inhibitors, such as PD-1/PD-L1 blockade, have demonstrated clinical benefits in subsets of patients with TNBC; however, their efficacy is highly dependent on pre-existing immune activation within the tumor microenvironment. Nanoparticle-based delivery systems have been developed to enhance drug retention and reduce off-target toxicity; however, many depend on complex fabrication procedures and non-readily available raw materials, limiting their scalability and translational feasibility.

Thermosensitive hydrogels represent a clinically attractive strategy for postoperative local therapy, as they can be injected in situ and form a drug depot at body temperature, thereby maintaining a high local drug concentration while minimizing systemic exposure. In this study, MND-ART-GEL integrates coordination-based MnUA immunomodulation, chemotherapeutic cytotoxicity, and sustained local delivery on a structurally simple platform. The hydrogel undergoes a sol–gel transition near physiological temperature and exhibits controlled in vivo degradation, supporting its feasibility for localized postoperative administration.

Importantly, the formulation combines oxidative stress induction, innate immune activation, and STAT3 pathway suppression, suggesting a multi-mechanistic approach to reshaping the tumor microenvironment after surgical resection. Given that the constituent materials are synthetically accessible and do not require complex nanofabrication or bioengineered components, this platform may offer advantages in terms of manufacturing simplicity and cost-effectiveness compared with highly engineered nanoparticle systems or biologic agents. These characteristics collectively support the translational potential of MND-ART-GEL as a postoperative anti-recurrence strategy for TNBC.

## Conclusion

In this study, MnUA microparticles were successfully synthesized via reverse microemulsion, wherein urate molecules were coordinated with Mn²^+^ through metal–ligand interactions. MnUA exhibited intrinsic immunostimulatory capability and served as a drug carrier by physically adsorbing the chemotherapeutic drug, DOX. Codelivery of DOX and ART within a Pluronic F127-based thermosensitive hydrogel resulted in the generation of a multifunctional platform—MND-ART-GEL.

Both in vitro and in vivo studies revealed that MND-ART-GEL exerts potent therapeutic efficacy in primary and postsurgical 4T1 TNBC mouse models. DOX and ART synergistically increased intracellular ROS levels, causing oxidative stress, mitochondrial damage, and subsequent tumor cell apoptosis and ICD. These changes enhanced the exposure of immunogenic markers (MHCI and Fas) to tumor cells. Simultaneously, MnUA functions as an immune adjuvant to promote the infiltration and maturation of DCs, while ICD further activates antigen presentation and T-cell responses. MND-ART-GEL also reduced PD-L1/PD-1 expression, alleviated T-cell exhaustion, and enhanced CD8^+^ T-cell–mediated cytotoxicity. Moreover, STAT3 inhibition by ART suppressed tumor invasion and metastasis.

This study introduces a novel application of MSU as an immune adjuvant, integrating chemotherapy, immunomodulation, and multitarget therapy to reshape the tumor immune microenvironment. The platform effectively inhibited tumor growth and recurrence, offering a promising therapeutic strategy for TNBC that is expected to overcome treatment resistance and improve patient outcomes.

## Supplementary Information

Below is the link to the electronic supplementary material.

Supplementary Material 1.

## Data Availability

All data analyzed during this study are included in this article.
