# Supplementary material for: MnUA–DOX–artesunate hydrogel remodels immunosuppressive tumor microenvironment and prevents postoperative recurrence in triple-negative breast cancer
Source: J Nanobiotechnology. 2026 Apr 18;24:511. doi: 10.1186/s12951-026-04381-7 (PMC13220432; doi:10.1186/s12951-026-04381-7)
Supplement: Supplementary file 1 — Supplementary Material 1. [file 12951_2026_4381_MOESM1_ESM.docx]

**MnUA–DOX–Artesunate Hydrogel Remodels Immunosuppressive Tumor Microenvironment and Prevents Postoperative Recurrence in Triple-negative Breast Cancer**

Keneng Cai^1^, Run Xia^1^, Mengyao Xu^1^, Wanying Chen^1^, Wenli Cai^1^, Xinyu Liu^1^, Tiantian Guo^1^, Weichi Jiang^1,2^, Chuyi Yu^1,2^, Jianjia Feng^1^, Chengli Ling^3^, Sheng Zhou^1^, Yinhuan Chen^2^, Changsheng Deng^1^, Qin Xu^1^, and Jianming Liang*^1,2^

^1^Artemisinin Research Center, Guangzhou University of Chinese Medicine, Guangzhou 510006, China.

^2^The First Afffliated Hospital of Guangzhou University of Chinese Medicine, Guangzhou 510405, China.

^3^Hunan academy of Chinese Medicine, Yuehua road, Yuelu district, Changsha 410013, China

Corresponding author:

Name: Jianming Liang, E-mail: liangjianming@gzucm.edu.cn.

**Materials.**

Sodium urate was purchased from Yuanye Biotechnology (Shanghai, China), and manganese chloride was obtained from Heowns Biochem Technologies (Tianjin, China). Cyclohexane and n-hexanol were acquired from Mairuida (Beijing, China). Triton X-100, Pluronic F127, Hoechst 33342, lipopolysaccharide (from E. coli O55:B5), and Alexa Fluor Plus 488-conjugated secondary antibody were purchased from Sigma-Aldrich (USA). Doxorubicin hydrochloride and artesunate were obtained from Melonepharma (Dalian, China). CCK-8 assay kits were from RARBIO (Guangzhou, China). RPMI-1640 medium, triple antibiotic solution, and fetal bovine serum (FBS) were provided by Procell (Wuhan, China) and Gibco (USA), respectively. DAPI was purchased from Macklin (Shanghai, China).

Annexin V-AF647 was from Elabscience (Wuhan, China). JC-10 probe and Z-DEVD-AFC were obtained from BIOBYING (Beijing, China) and GLPBIO (USA), respectively. Reactive oxygen species detection kits and BCA protein assay kits were from Beyotime (Shanghai, China).

Matrigel matrix and Transwell chambers were purchased from Corning (USA). Paraformaldehyde (4%) was from Biosharp (Beijing, China). Crystal violet solution (1%) was obtained from Beekman (Changde, China). Murine GM-CSF was from PeproTech (USA).

H&E staining kit, neutral balsam, TUNEL assay kit, proteinase K, anti-Ki67 antibody, and DAB substrate kit were all provided by Servicebio (Wuhan, China). ELISA MAX Deluxe kits for mouse IL-6, TNF-α, and IFN-γ were purchased from BioLegend (USA), and ELISA plates (F96 MAXISORP NUNC-IMMUNO PLATE) were from Thermo Fisher Scientific (USA).

p-STAT3 Tyr705 (#YP0251), p-STAT3 Ser727 (#YP0250), p-JAK2 (#YP0155), JAK2 (#YM8330), p-STING (#YP1518), p-TBK1 (#YM8674) antibodies were purchased from ImmunoWay (USA). CALR (#E-AB-10352) and STAT3 (#E-AB-32982) antibodies were purchased from Elabscience (Wuhan, China). Anti-β-actin (#ZB15001-HRP-100) was obtained from Servicebio (Wuhan, China), and HRP goat anti-rabbit IgG (#A21020) from Abbkine (Wuhan, China). Antibodies against HMGB1 (#bs-0664R) and Cy5-labeled goat anti-rabbit IgG secondary antibodies (#bs-0295G-Cy5) were from Bioss (Beijing, China). Alexa Fluor Plus 488-labeled goat anti-rabbit IgG secondary antibodies (#XH353659) were from Invitrogen (USA).

Flow cytometry antibodies, including APC anti mouse MHCI (#17-5958-82), Alexa Fluor 488 anti mouse Fas (#53-0951-82) were obtained from Invitrogen (USA). APC anti mouse CD11c (#117310), PE anti mouse CD86 (#105008) were obtained from BioLegend (USA). EV450 anti mouse CD45 (#E-AB-F1136Q), FITC anti mouse F4/80 (#E-AB-F0995C), ER780 anti mouse CD11b (E-AB-F1081S), PE anti mouse CD206 (#E-AB-F1135D), APC anti mouse CD86 (#E-AB-F0994E), EV450 anti mouse CD11c (#E-AB-F0991Q), PerCP/Cy5.5 anti mouse MHCII (#E-AB-F0990J), PE anti mouse PD-L1 (#E-AB-F1132D), APC/Cy7 anti mouse CD3 (#E-AB-F1013N), FITC anti mouse CD8a (#E-AB-F1104C), APC anti mouse CD4 (#E-AB-F1097E), PE/Cy7 anti mouse PD-1 (#E-AB-F1131H) were obtained from Elabscience (Wuhan, China).

**Instruments.**

A Smart-S30 ultrapure water purification system was obtained from Heal Force (Shanghai, China). Magnetic stirring was performed using an HMS-1 stirrer from Shanghai Huxi Industrial Co., Ltd. High-speed centrifugation was carried out using a TGL-16M centrifuge (Xiangyi, Hunan, China). Constant-temperature shaking incubations were performed using an HZQ-F100 incubator (Peiying, Suzhou, China).

Rotary evaporation was conducted using an RE-2000A evaporator (Xiande, Shanghai, China). Weighing was performed with a Practum electronic balance (Sartorius, Germany). Cell culture procedures were performed in a BioBase laminar flow hood (Shandong, China) and a BB150 CO₂ incubator (Thermo Fisher Scientific, USA).

Ultrasonication was conducted using a PS-100A cleaner (JieKang, Dongguan, China) and a JY92-IIN cell disruptor (Ningbo Scientz, China). UV-Vis absorbance was measured using a Varioskan LUX microplate reader (Thermo Fisher Scientific, USA). HPLC analysis was performed using the Ultimate 3000 system (Thermo Fisher Scientific, USA) and LCMS-2050 system (Shimadzu Co., Kyoto, Japan), and SEM imaging was done with the Phenom ProX system (Thermo Fisher Scientific, USA).

Freeze-drying was performed using an ALPHA1-2LD lyophilizer (Martin Christ, Germany). FTIR spectra were acquired with a Cary 630 spectrometer (Agilent, USA), and elemental analysis was performed using the iCAP Qnova Series ICP-MS (Thermo Fisher Scientific, USA). Protein separation and detection used a Mini-PROTEAN Tetra electrophoresis system and PowerPac power supply (Bio-Rad, USA), and chemiluminescence imaging was performed with the Amersham ImageQuant 800 system (Cytiva, Sweden).

Fluorescence microscopy was conducted using an upright microscope (BX53F, Olympus, Japan), and flow cytometry was performed on a BD LSRFortessa cytometer (USA). RNA integrity was assessed using a NanoDrop 2000 spectrophotometer and Bioanalyzer 2100 system (Agilent, USA). Tissue grinding was performed with a KZ-III-F homogenizer (Servicebio, Wuhan, China).

Histological processing was carried out using a Donatello tissue processor (DIPATH, Italy), JB-P5 embedding center (Junjie Electronics, Wuhan, China), RM2016 rotary microtome (Leica, Germany), and KD-P section spreading machine (Kedi, Jinhua, China). Slide baking was done in a GFL-230 oven (Laibori, Tianjin, China), and digital scanning was completed with a KF-FL-005 scanner (Jiangfeng, Ningbo, China).


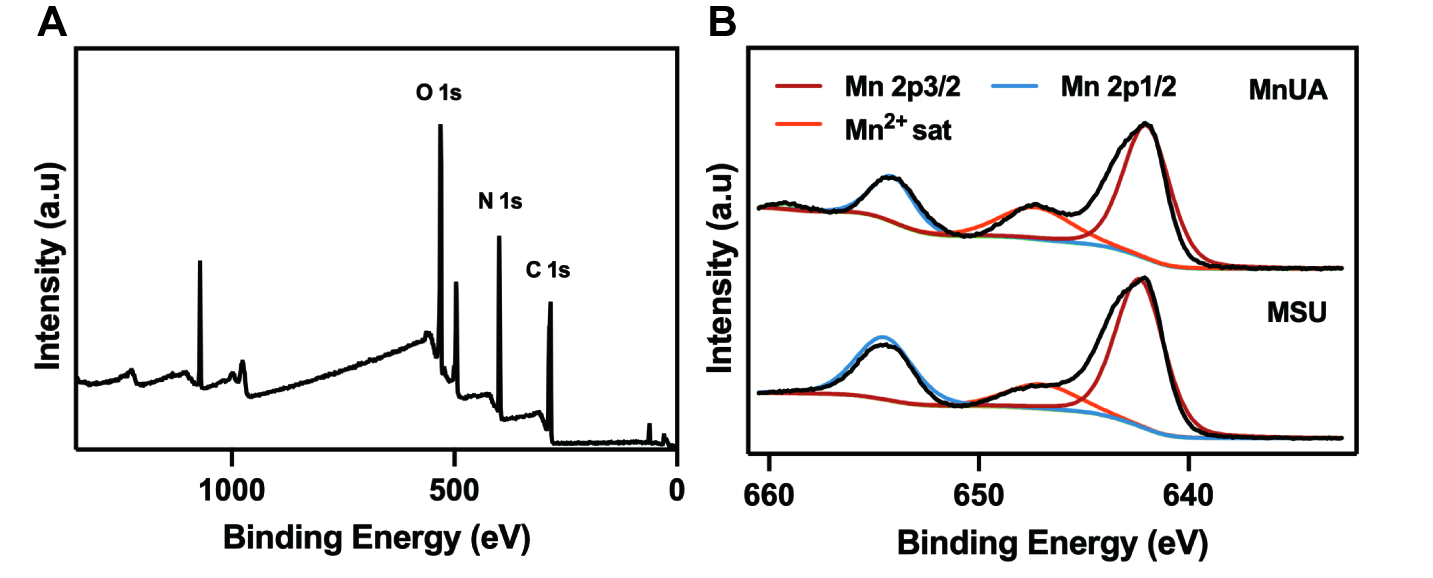


**Figure S1.** (A) XPS survey spectrum of MSU. (B) Mn 2p spectra of MnUA and MSU.


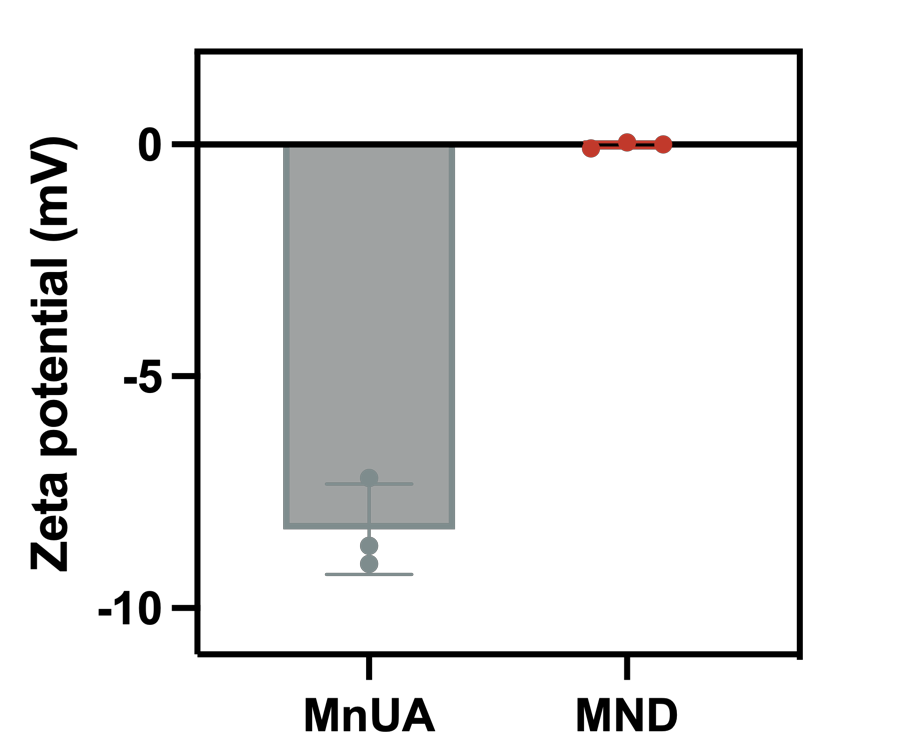


**Figure S2.** Zeta potential of MnUA and MND (n=3).


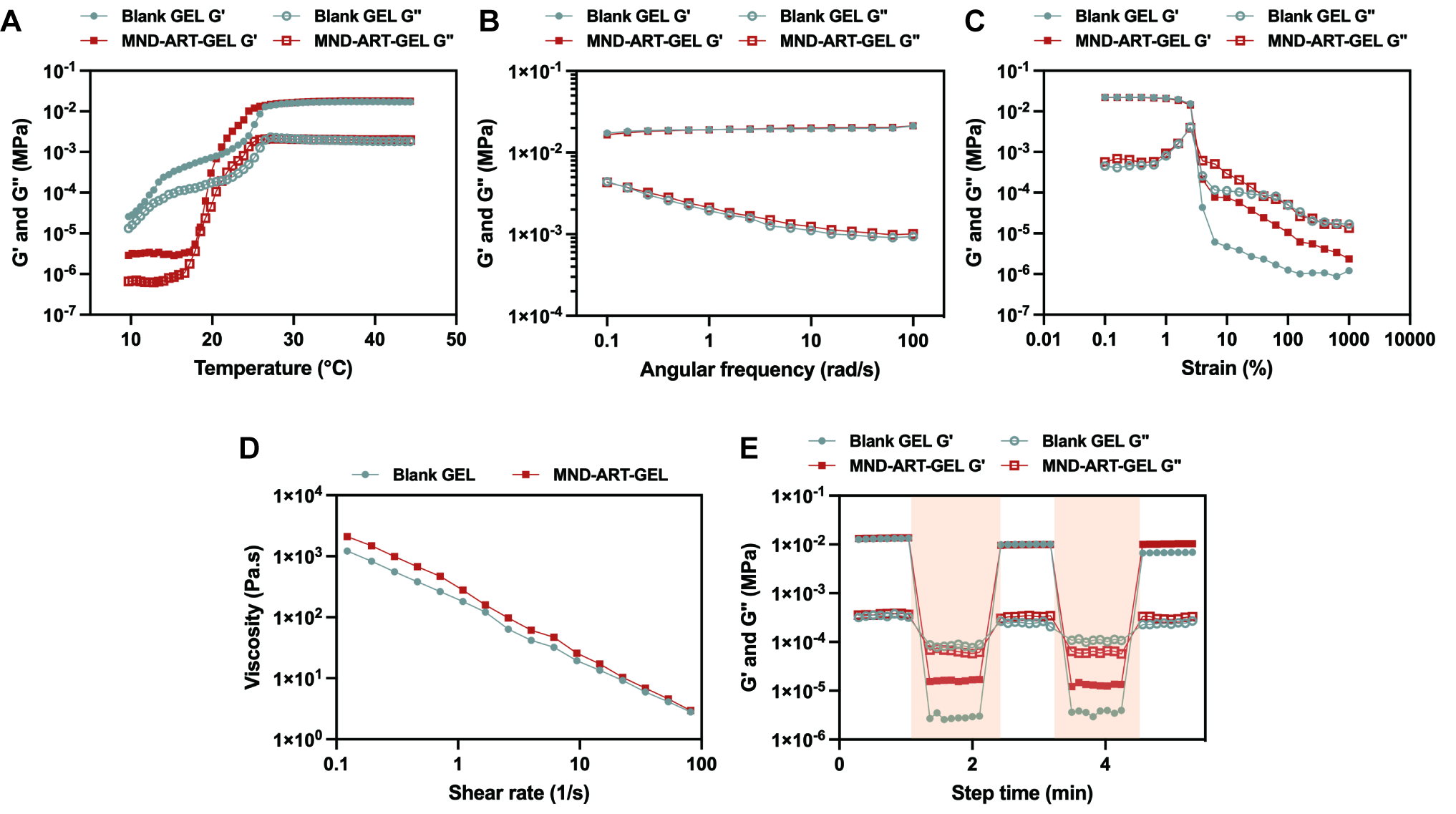


**Figure S3.** Rheological properties of Blank GEL and MND-ART-GEL. (A) Temperature sweeps of Blank GEL and MND-ART-GEL. (B) Frequency sweeps of Blank GEL and MND-ART-GEL. (C) Strain sweeps of Blank GEL and MND-ART-GEL. (D) Shear rate–dependent viscosity of Blank GEL and MND-ART-GEL. (E) Cycling strain sweeps of Blank GEL and MND-ART-GEL.


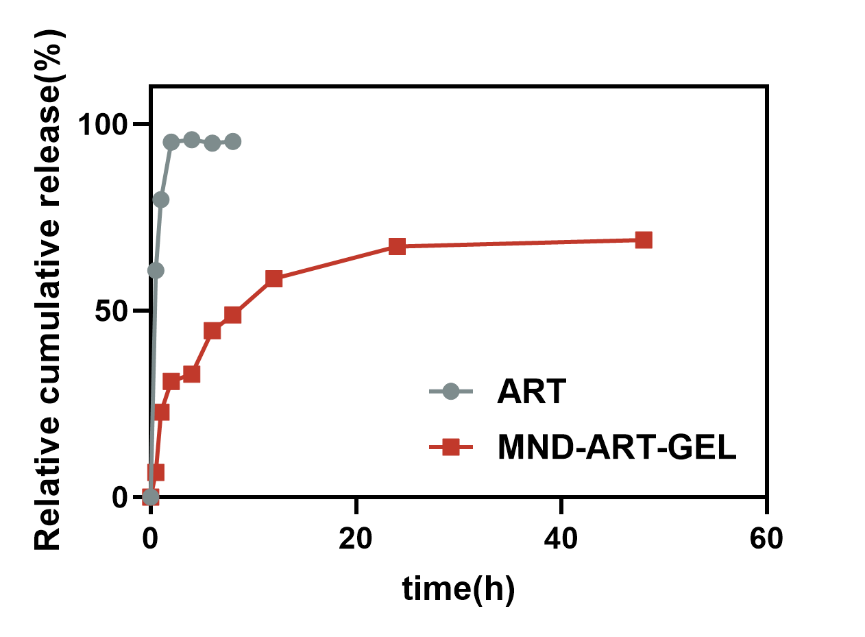


**Figure S4.** Cumulative in vitro release profiles of ART (n = 3).


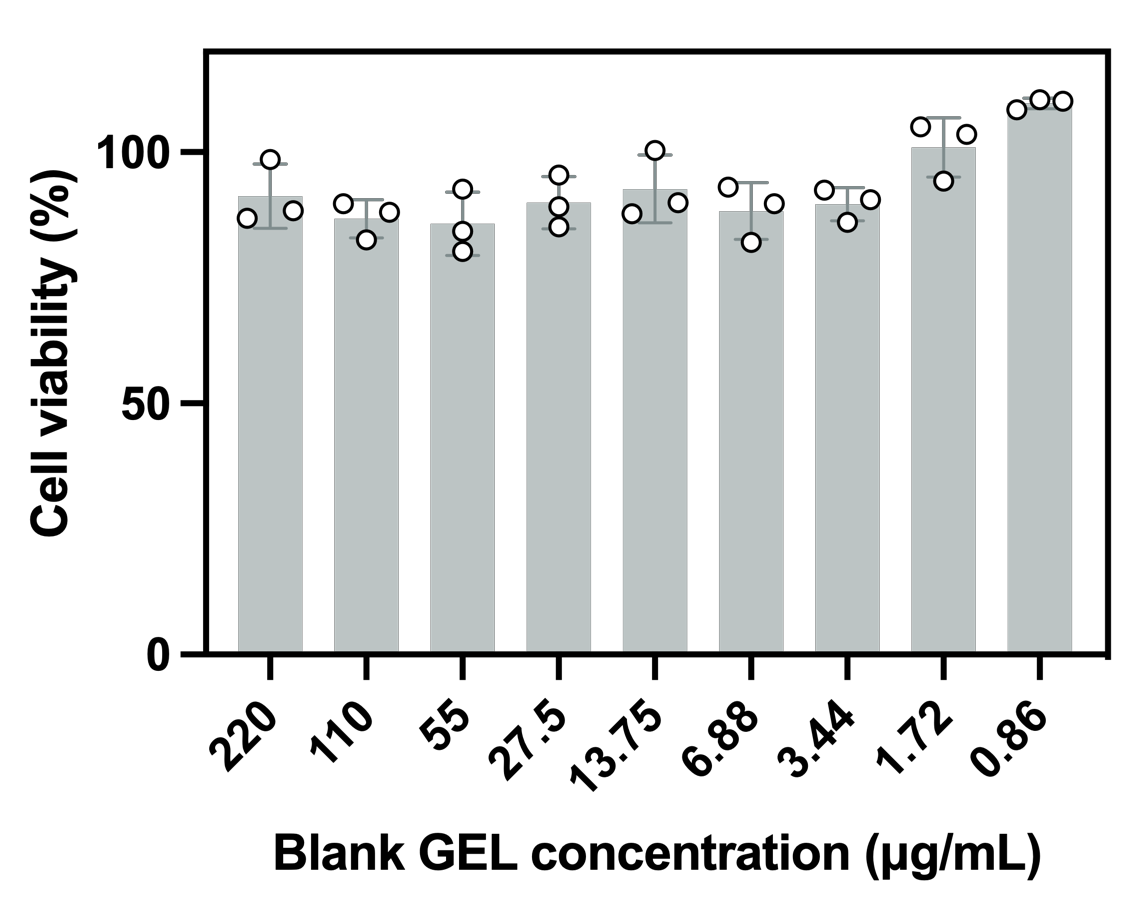


**Figure S5.** Cell viability of 4T1 cells treated with Blank GEL with respective concentrations (n = 3).


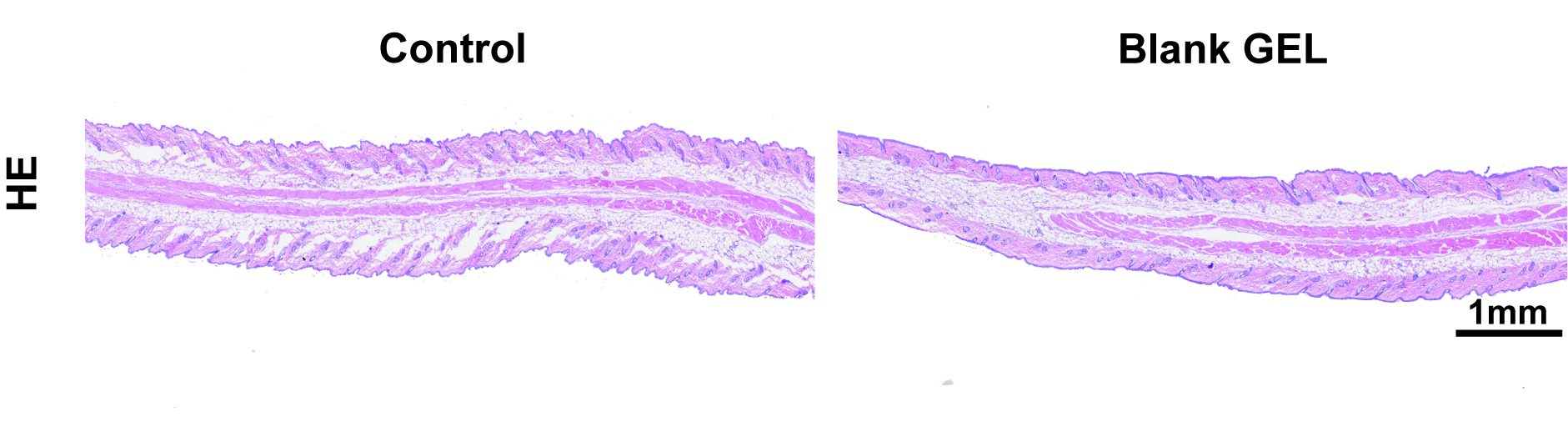


**Figure S6.** H&E staining images of skin tissues at the injection sites.


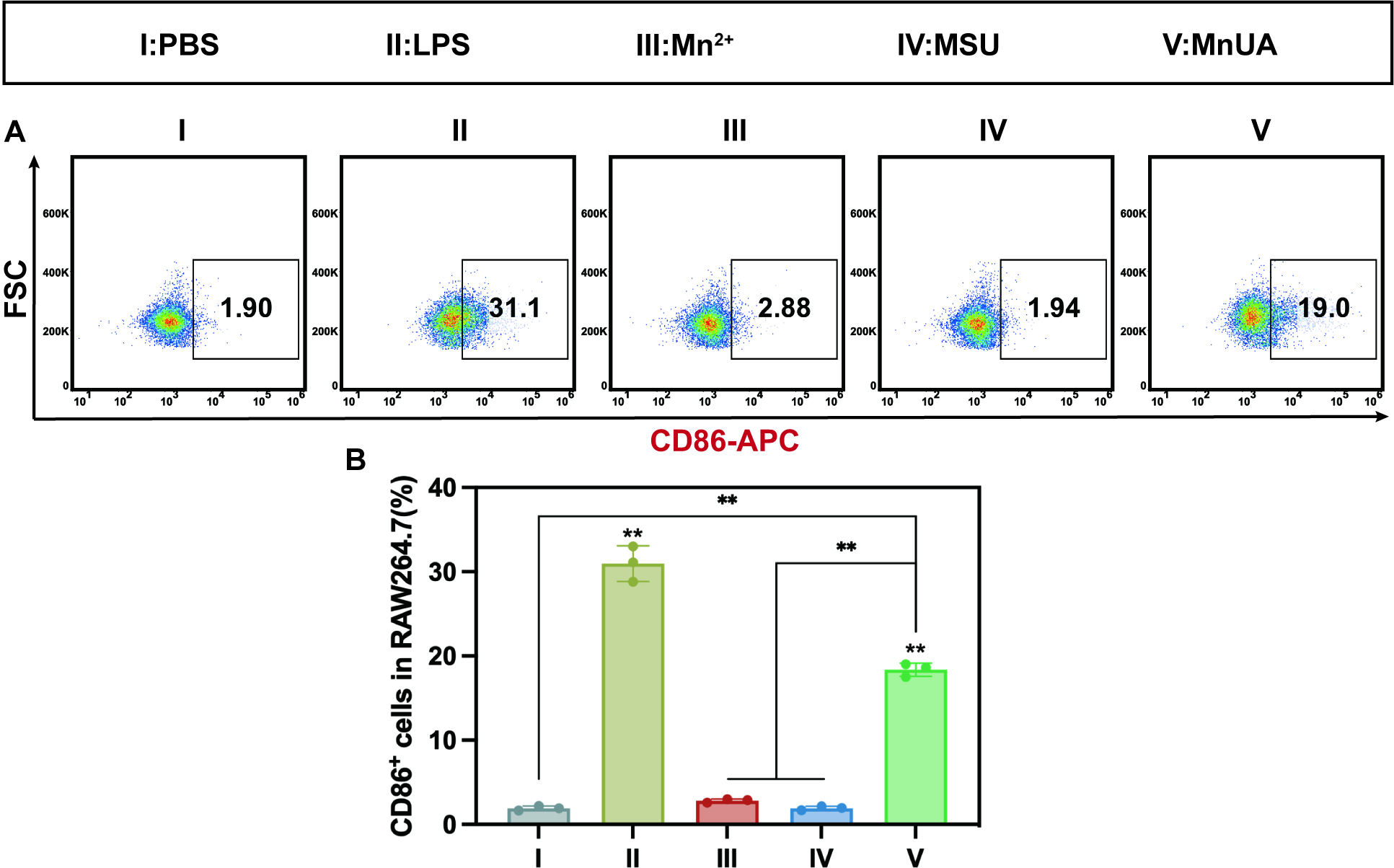


**Figure S7.** Representative flow cytometry scatter plots (A) and quantitative analysis (B) of M1 polarization in RAW264.7 cells (CD86^+^ cells%, n = 3). Statistical annotations: *P < 0.05, **P < 0.01 vs. PBS group; ns, not significant.


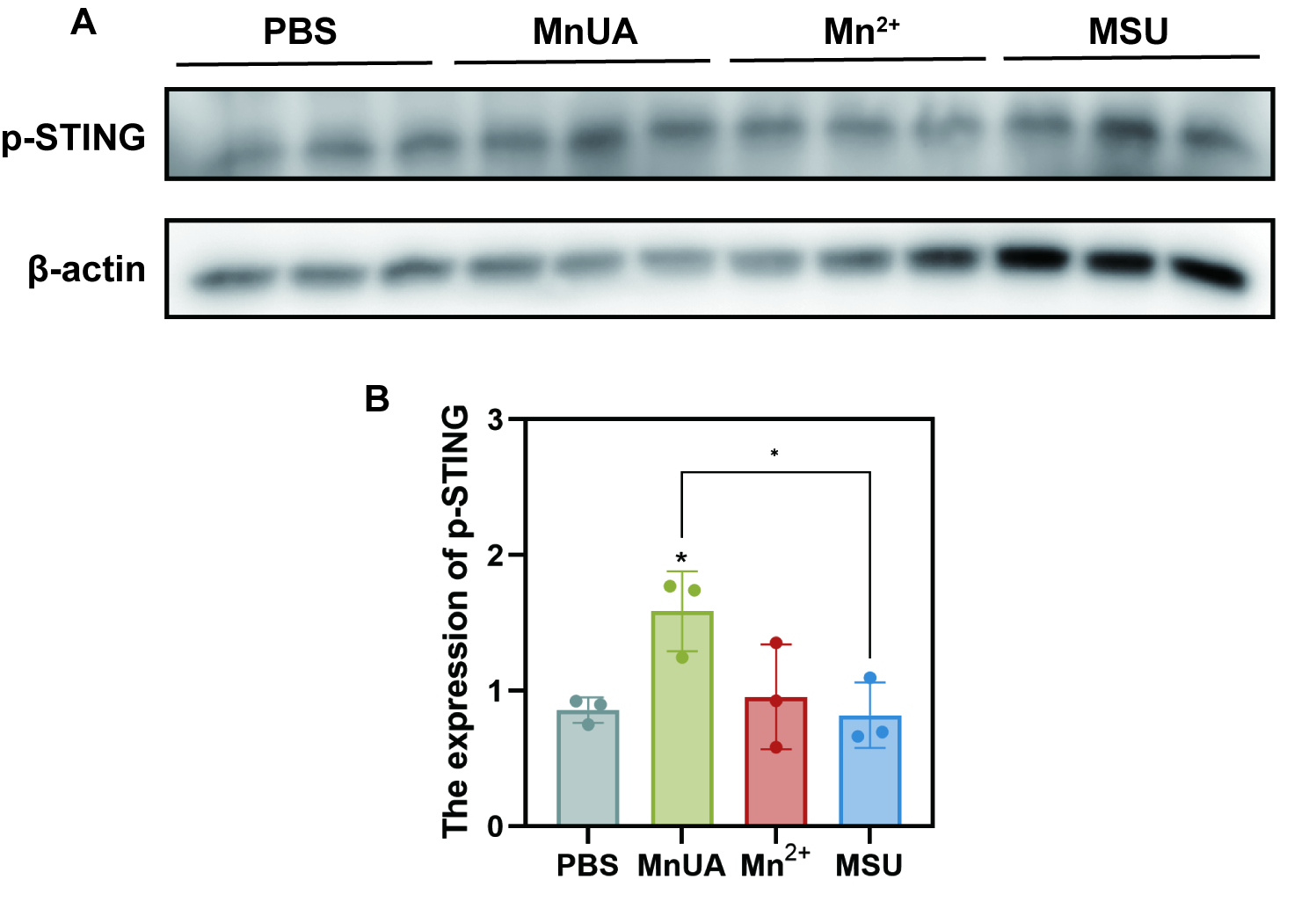


**Figure S8.** Representative western blots (A) with relative quantifications (B) of p-STING in BMDCs with drugs treated (n = 3). Statistical annotations: *P < 0.05, **P < 0.01 vs. PBS group; ns, not significant.

**
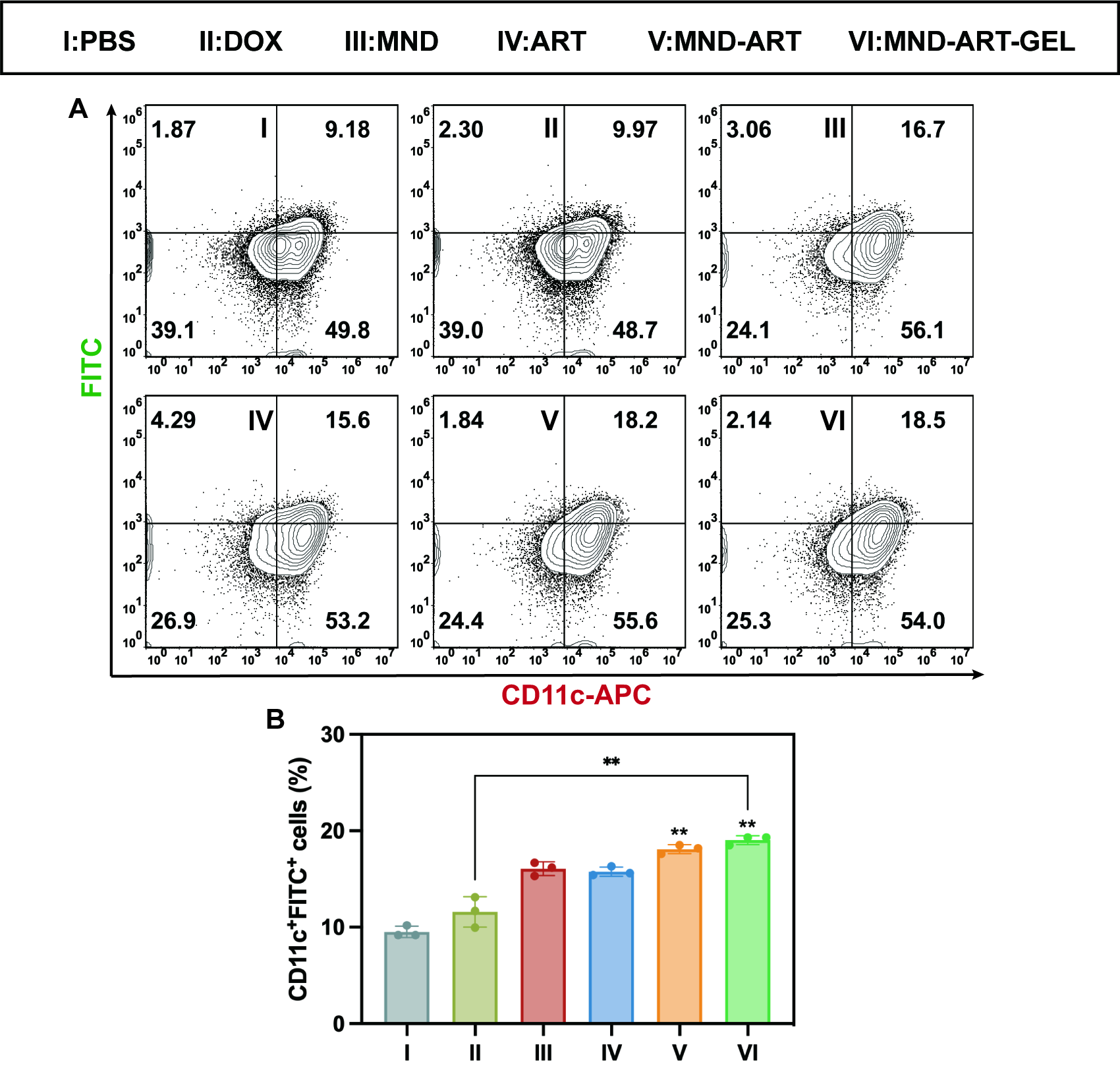
**

**Figure S9.** Representative flow cytometry scatter plots (A) and quantitative analysis (B) of BMDCs Phagocytosis Experiment (CD11c^+^FITC^+^ cells%, n = 3). Statistical annotations: *P < 0.05, **P < 0.01 vs. PBS group; ns, not significant.


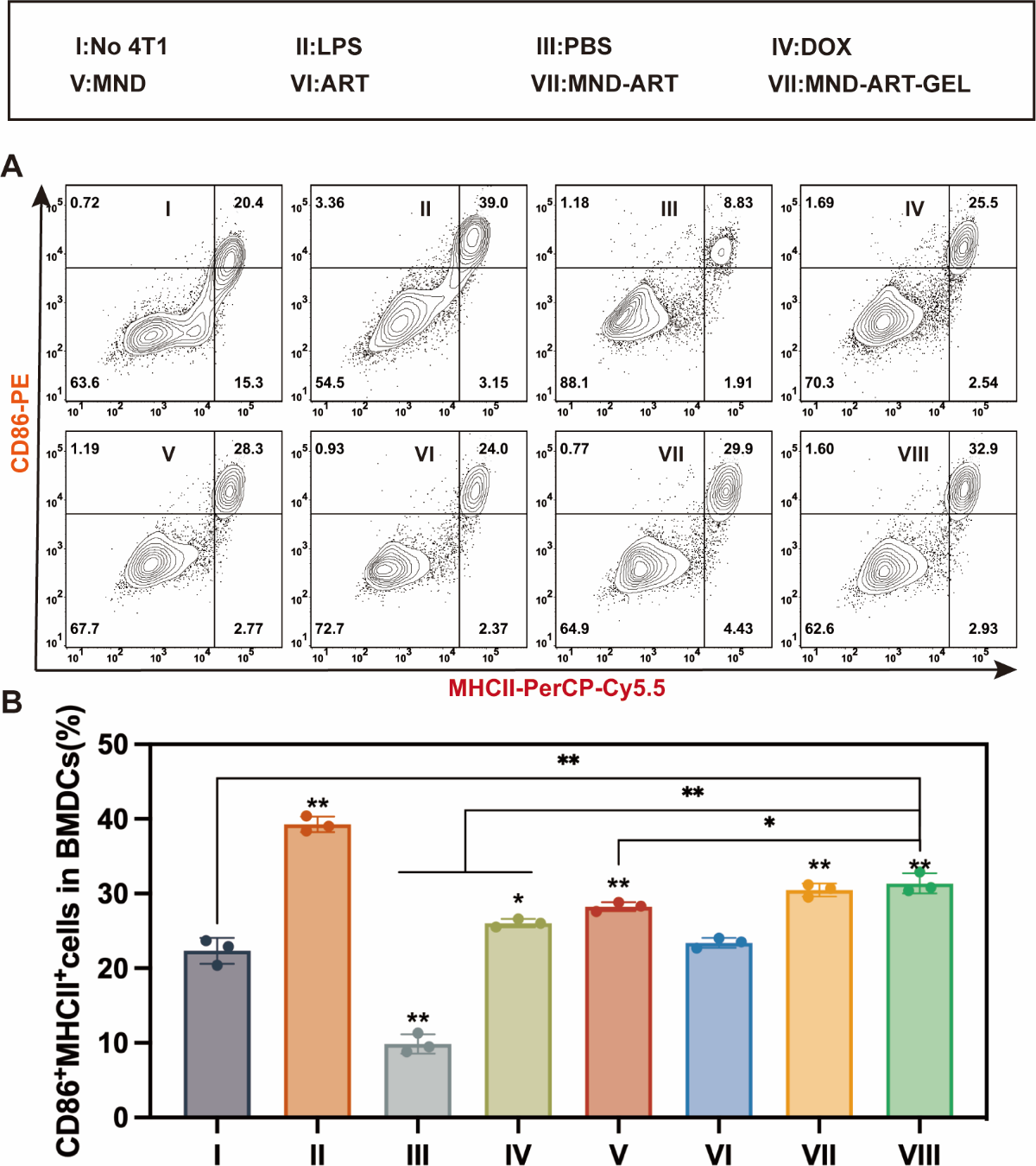


**Figure S10.** Co-culture assay of tumor cells and BMDCs. (A) Representative flow cytometry plots showing the maturation of BMDCs. (B) Quantification of mature BMDCs (CD86^+^MHCII^+^ cells, %, n = 3). Statistical annotations: *P < 0.05, **P < 0.01 vs. PBS group; ns, not significant.


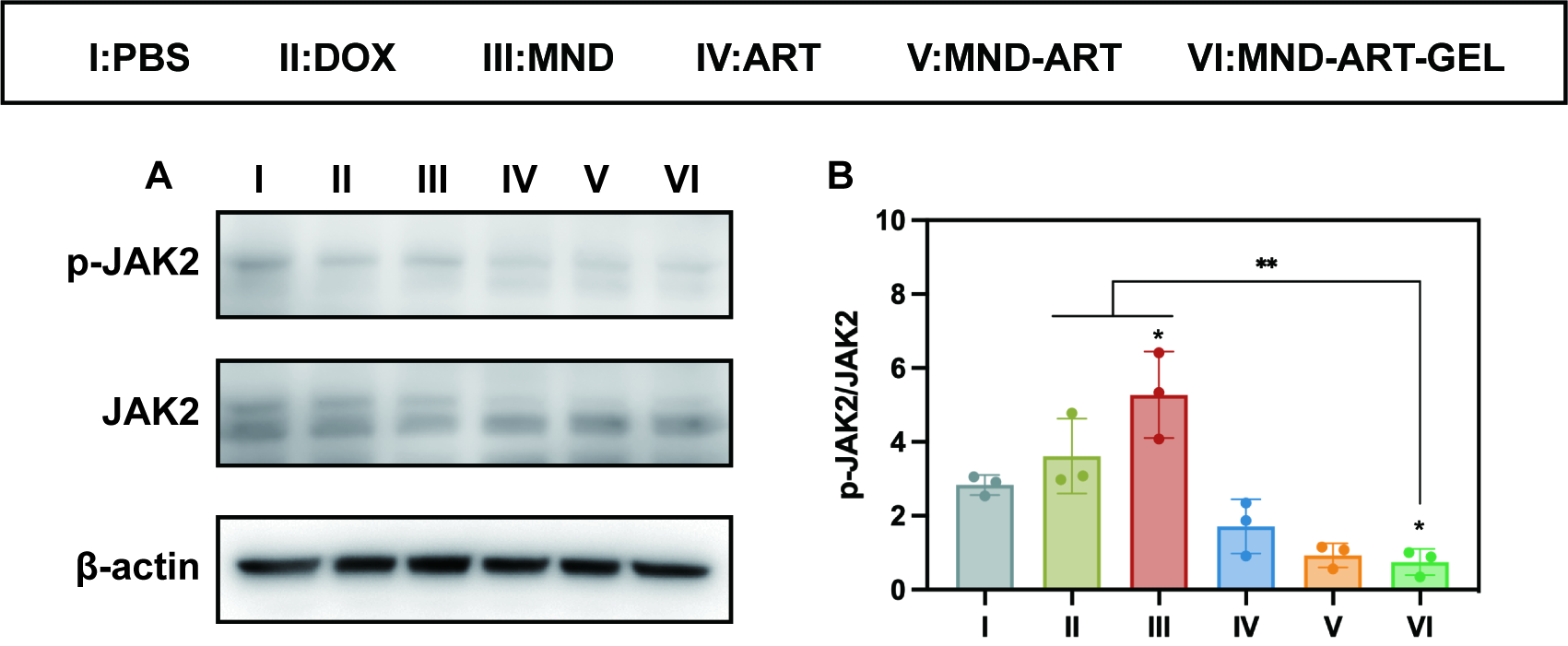


**Figure S11.** Representative western blots (A) with relative quantifications (B) of p-JAK2 in 4T1 cells with drugs treated (n = 3). Statistical annotations: *P < 0.05, **P < 0.01 vs. PBS group; ns, not significant.


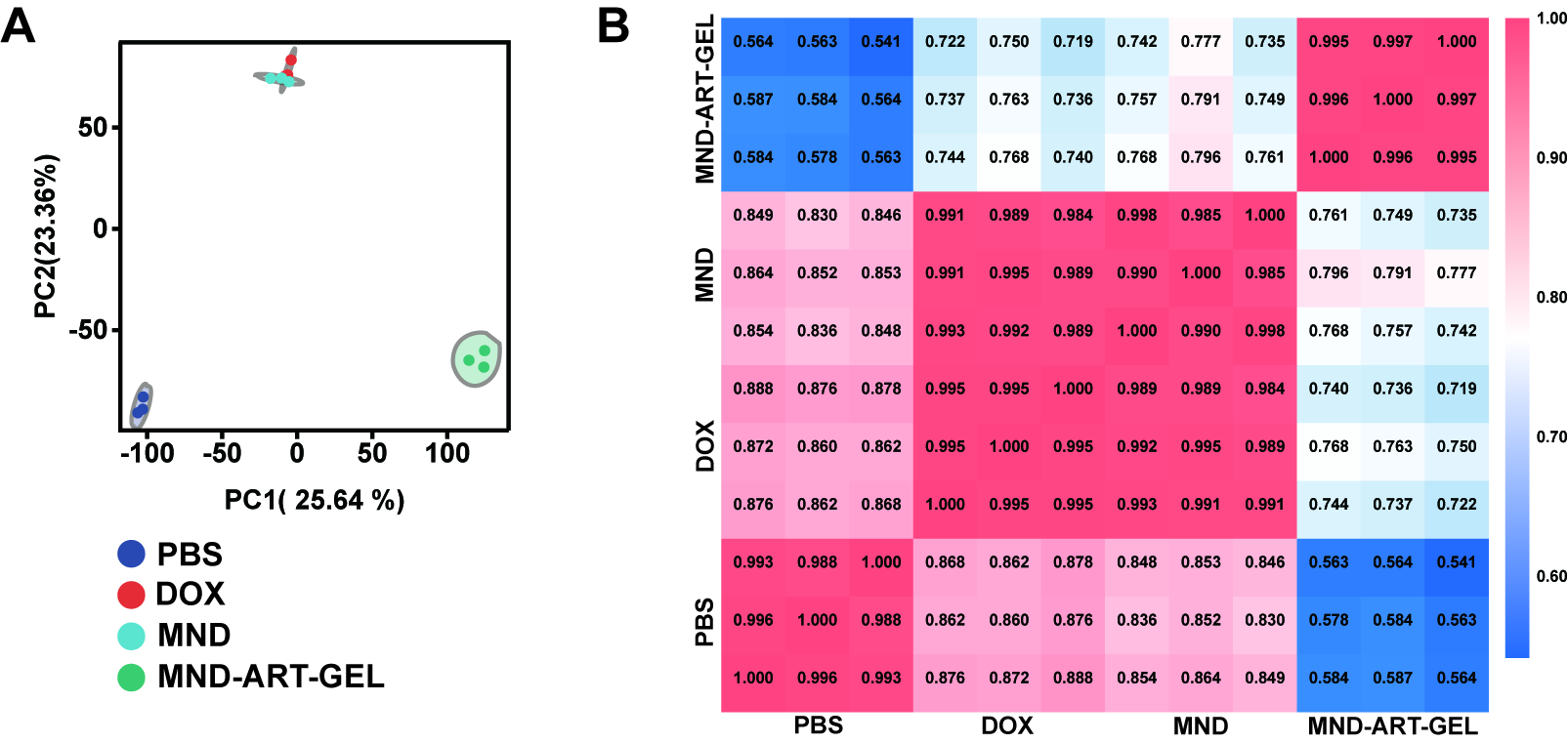


**Figure S12.** (A) Principal component analysis (PCA) of transcriptomic profiles. (B) Heatmap of sample correlation analysis.


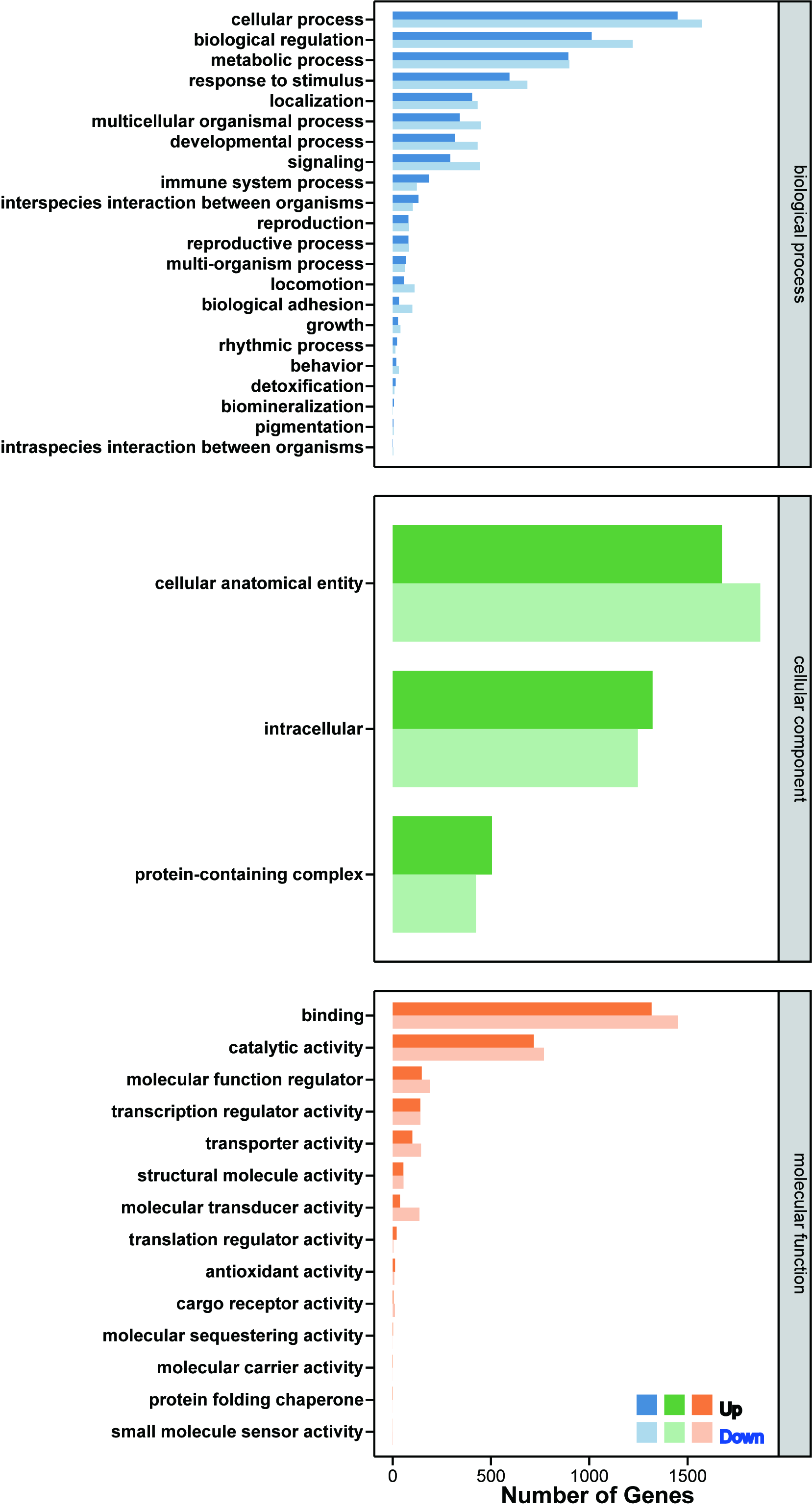


**Figure S13.** GO classification of differentially expressed genes between PBS and MND-ART-GEL groups.


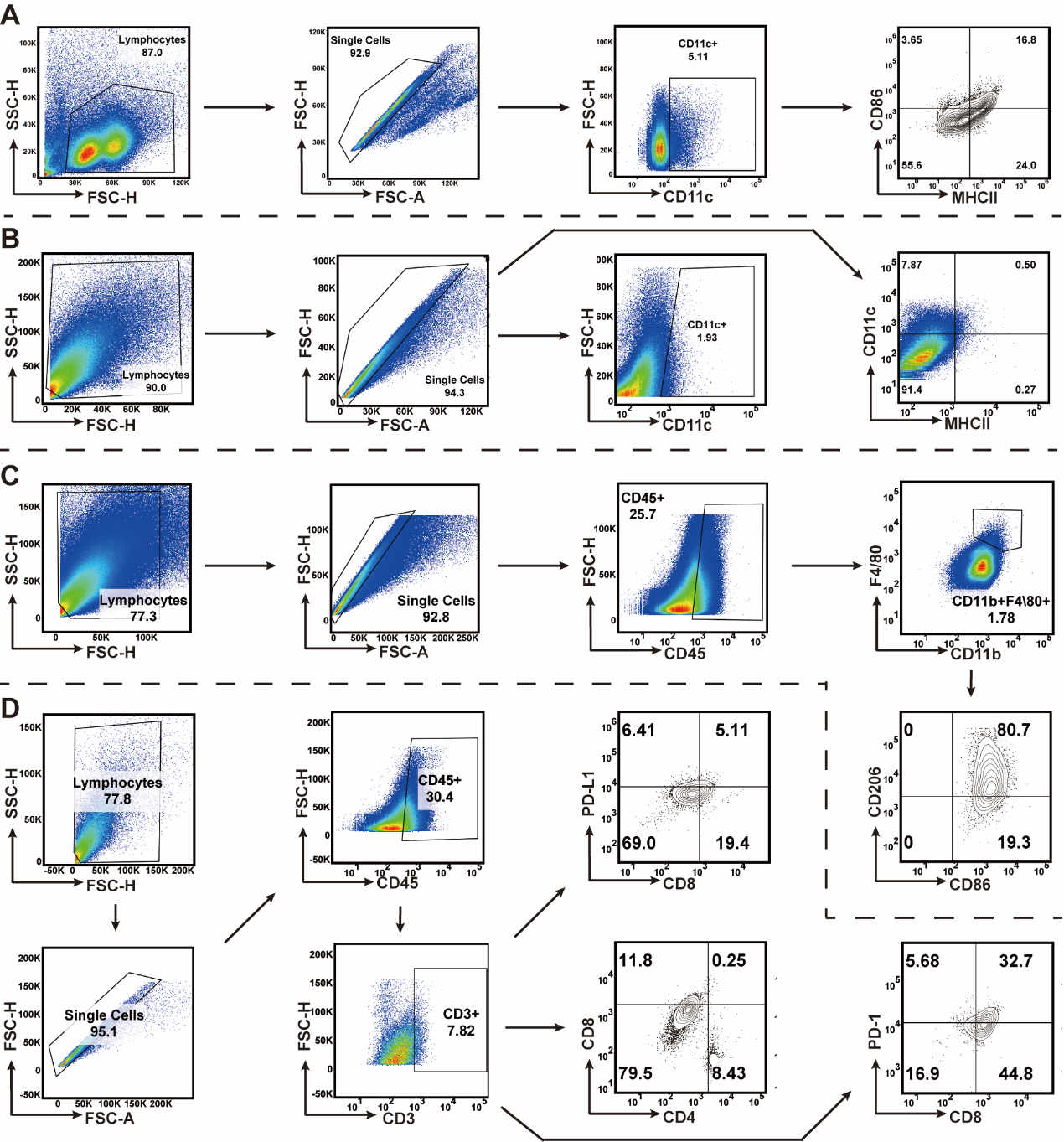


**Figure S14.** (A) Gating strategy for DCs in tumor-draining lymph nodes (TDLNs). (B) Gating strategy for DCs in tumor tissue. (C) Gating strategy for tumor-associated macrophages (TAMs) in tumor tissue. (D) Gating strategy for T cells in tumor tissue.


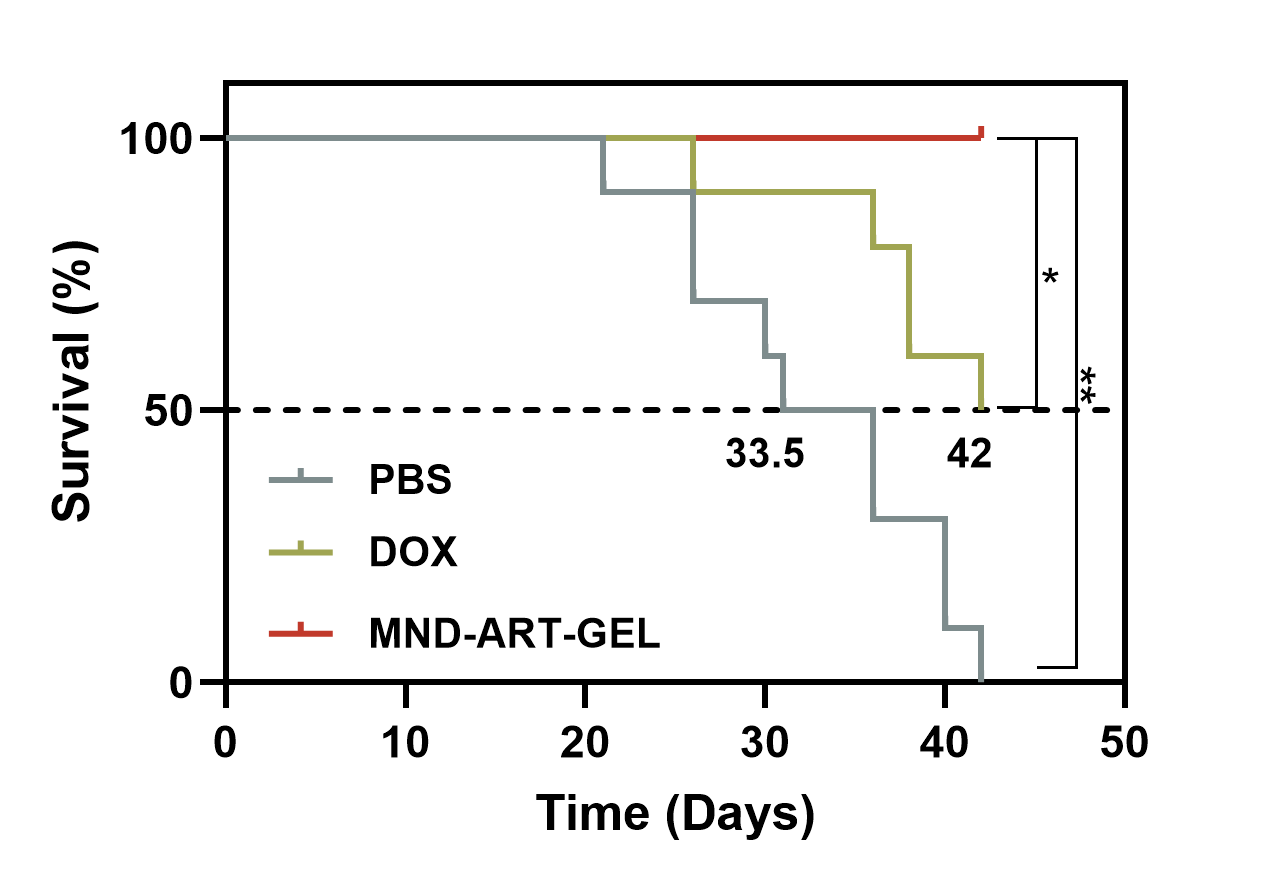


**Figure S15.** Survival analysis of breast cancer recurrence model mice treated with different formulations (n=10). Statistical annotations: *P < 0.05, **P < 0.01 vs. PBS group; ns, not significant.


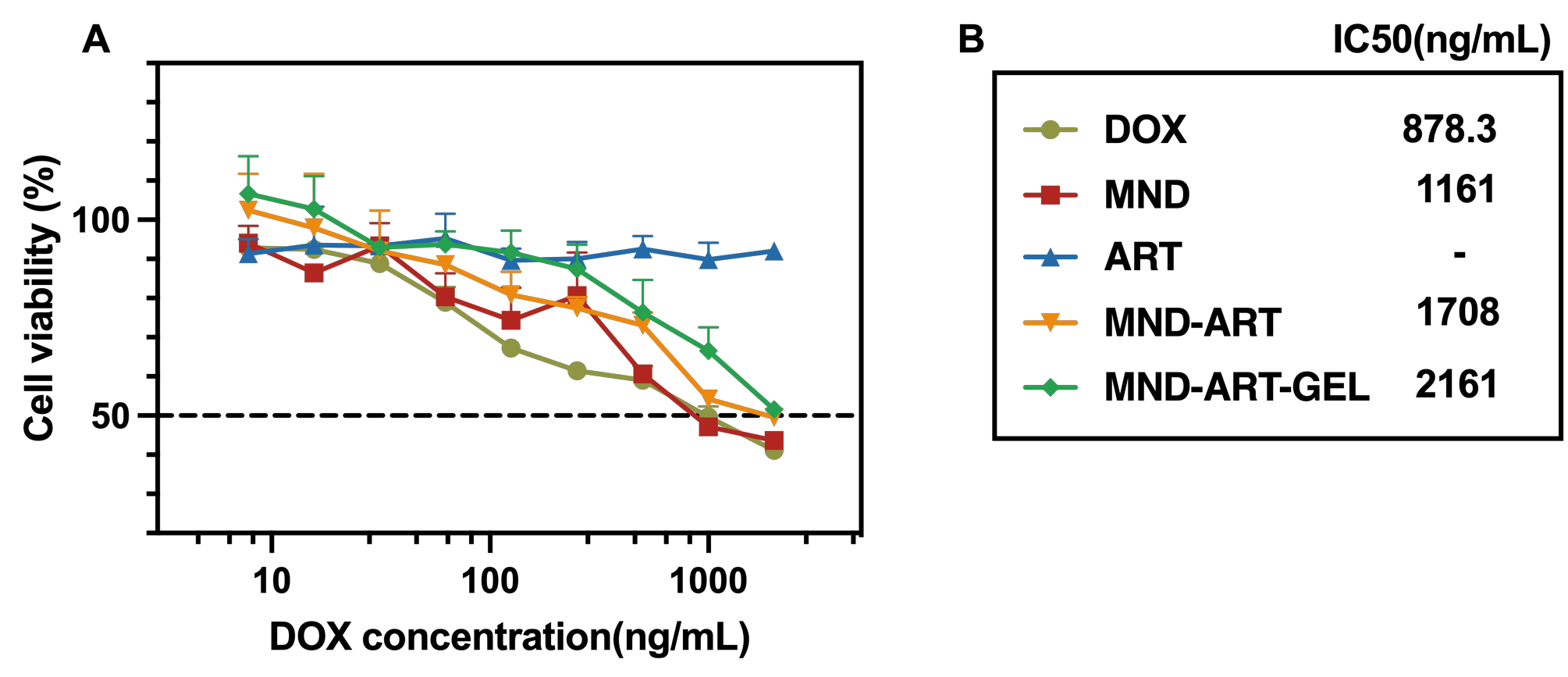


**Figure S16.** Cell viability curves (A) and IC₅₀ values (B) of HL-1 cells treated with different groups (n = 3).


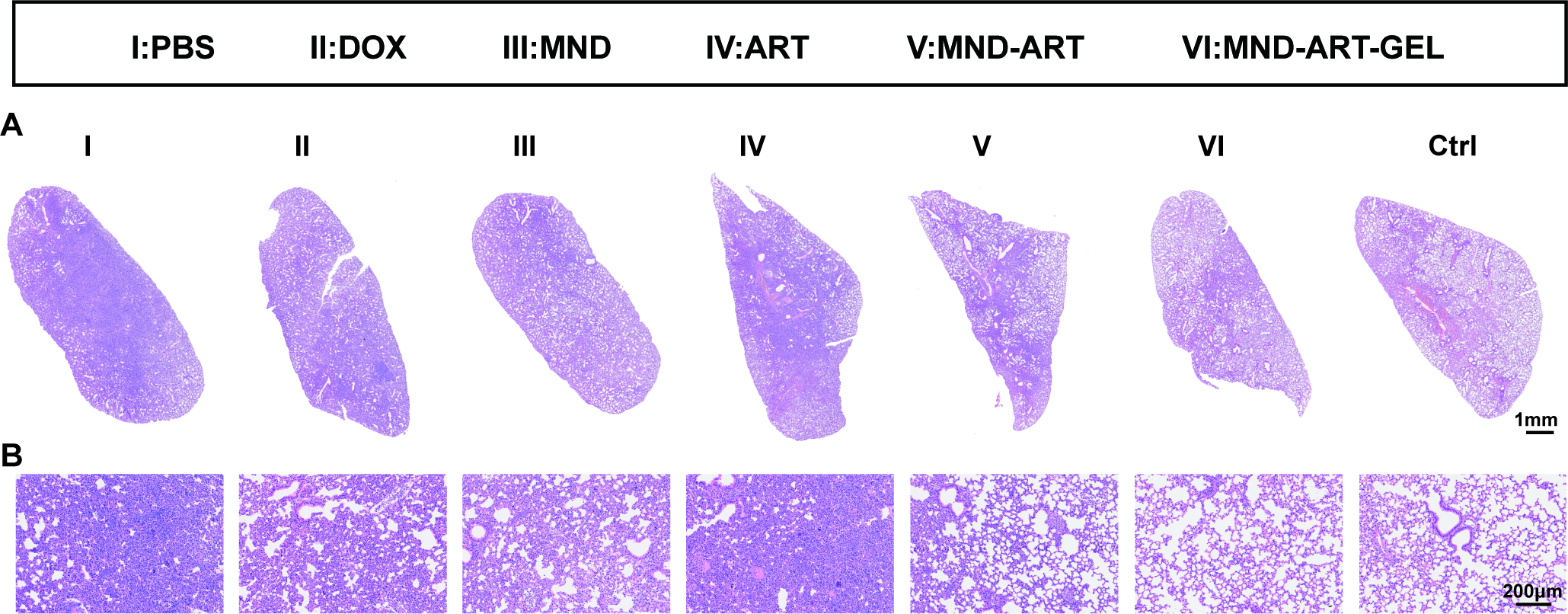


**Figure S17.** Representative digital photographs and bright-field HE-stained images of lung sections in different treatment groups.


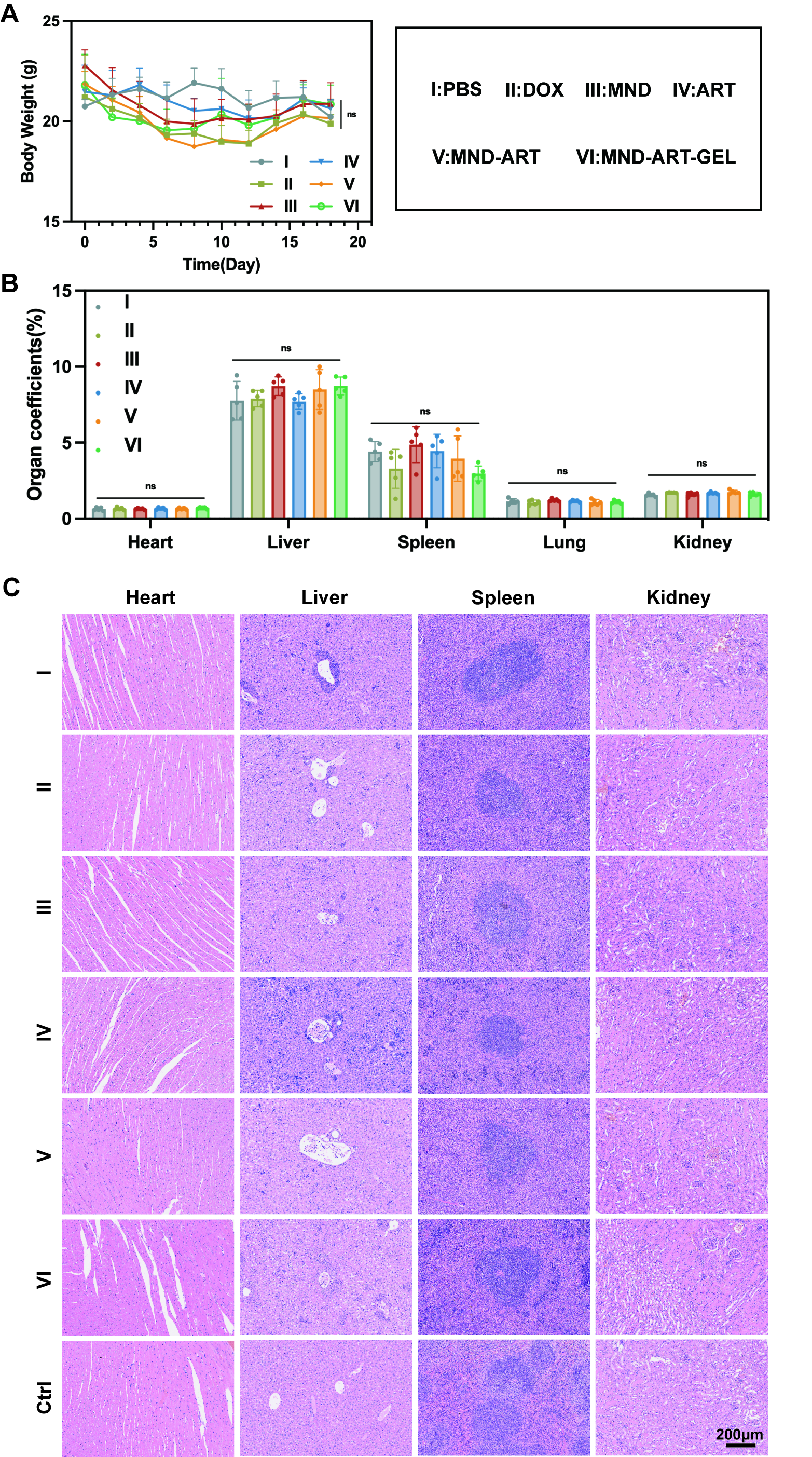


**Figure S18.** (A) Body weight curves of mice during treatment (n = 5). (B) Organ coefficient analysis at the endpoint (n = 5). (C) H&E staining images of major organs. Statistical annotations: *P < 0.05, **P < 0.01 vs. PBS group; ns, not significant.

**
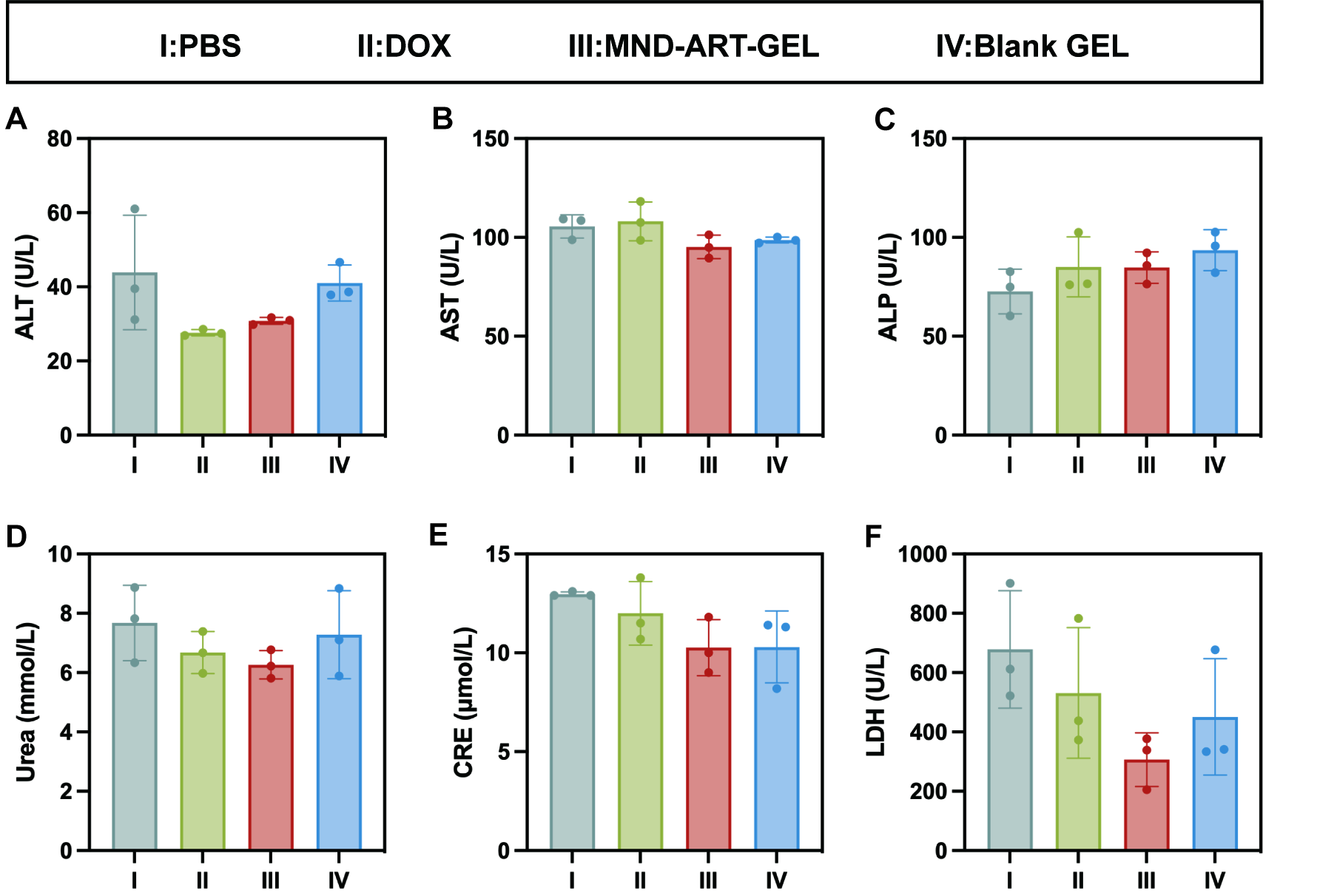
**

**Figure S19.** Blood biochemistry analysis in healthy mice after subcutaneous injection (n = 3).


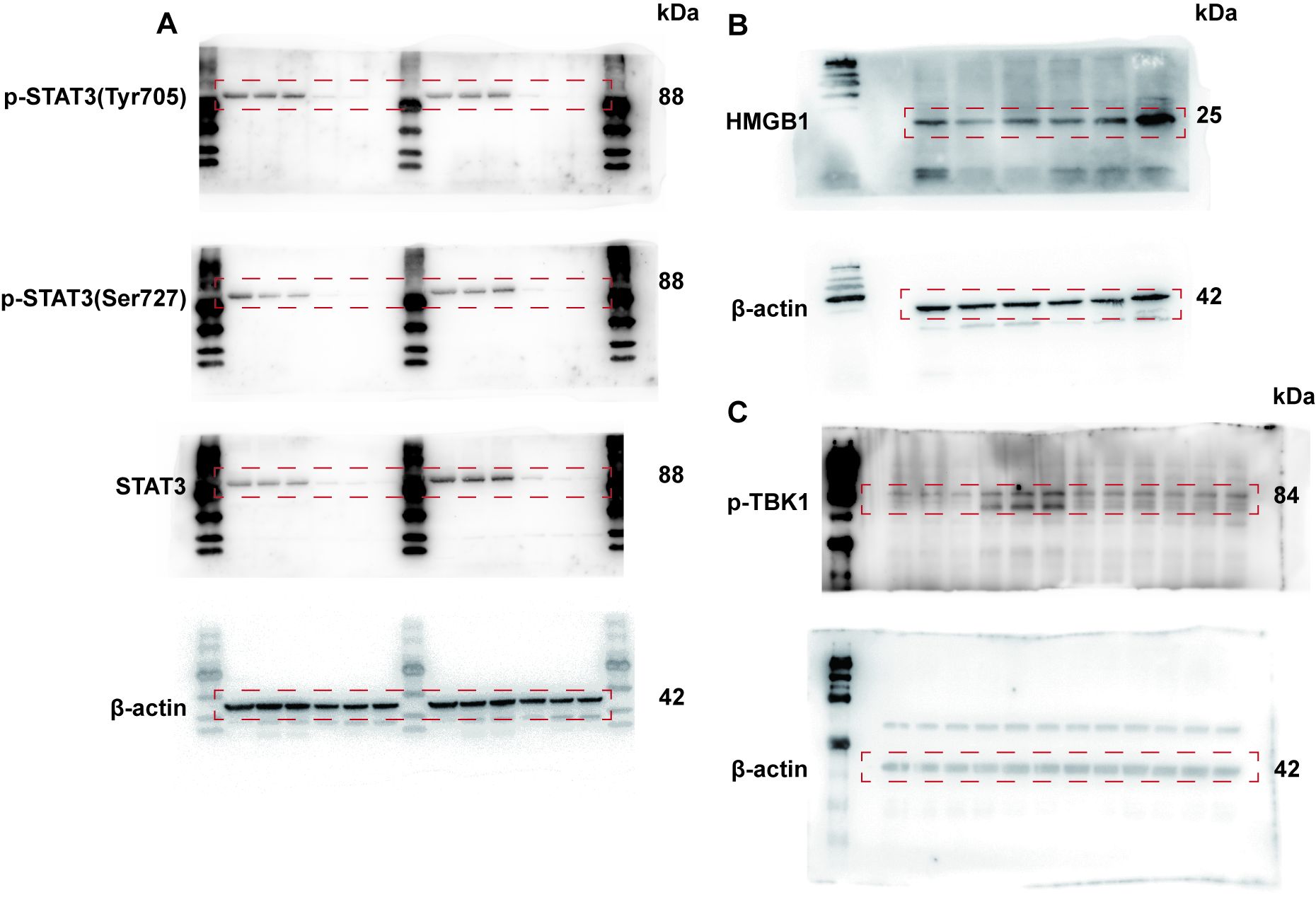


**Figure S20.** Representative original Western blot images of p-STAT3, STAT3, HMGB1, and p-TBK1.
